# AlphaDIA enables DIA transfer learning for feature-free proteomics

**DOI:** 10.1038/s41587-025-02791-w

**Published:** 2025-10-21

**Authors:** Georg Wallmann, Patricia Skowronek, Vincenth Brennsteiner, Mikhail Lebedev, Marvin Thielert, Sophia Steigerwald, Mohamed Kotb, Oscar Despard, Tim Heymann, Xie-Xuan Zhou, Maximilian T. Strauss, Constantin Ammar, Sander Willems, Magnus Schwörer, Wen-Feng Zeng, Matthias Mann

**Affiliations:** 1 Proteomics and Signal Transduction, Max Planck Institute of Biochemistry, Martinsried, Germany; 2 Proteomics Program, Novo Nordisk Foundation Center for Protein Research, Faculty of Health and Medical Sciences, University of Copenhagen, Copenhagen, Denmark

**Keywords:** Proteomics, Proteome informatics

## Abstract

The scale of data generated for mass-spectrometry-based proteomics and modern acquisition strategies poses a challenge to bioinformatic analysis. Search engines need to make optimal use of the data for biological discoveries while remaining statistically rigorous, transparent and performant. Here we present alphaDIA, a modular open-source search framework for data-independent acquisition (DIA) proteomics. We developed a feature-free identification algorithm that performs machine learning directly on the raw signal and is particularly suited for detecting patterns in data produced by time-of-flight instruments. Benchmarking demonstrates competitive identification and quantification performance. While the method supports empirical spectral libraries, we propose a search strategy named DIA transfer learning that uses fully predicted libraries. This entails continuously optimizing a deep neural network for predicting machine-specific and experiment-specific properties, enabling the generic DIA analysis of any post-translational modification. AlphaDIA provides a high performance and accessible framework running locally or in the cloud, opening DIA analysis to the community.

## Abstract (web summary)

An open-source platform for data-independent acquisition proteomics adapts predicted libraries to experimental settings.

## Main

Proteomics entails the study of key players of life—proteins—and their translation, composition of isoforms, post-translational modification (PTM) and degradation^1^. As proteomes are composed of thousands of different proteoforms, which produce hundreds of thousands of peptides in bottom-up proteomics, handling complexity is central to mass spectrometry (MS)-based proteomics acquisition and bioinformatic analysis.

Until recently, data-dependent acquisition (DDA) was the acquisition method of choice. The direct relationship between selected precursors and relatively pure fragmentation spectra, combined with its mature ecosystem of search engines, results in confident peptide identifications^2–5^. It has therefore establised itself even in the most challenging applications like complex patterns of PTMs or the interpretation of interprotein crosslinks^6,7^. Yet, selecting only a single peptide at a time comes at the cost of increased data acquisition time and stochastic sampling of precursors across liquid chromatography (LC)–MS runs^8^.

In contrast to DDA, data-independent acquisition (DIA) allows the selection of multiple peptides in parallel, originally in the form of cycles of fixed-width, relatively wide selection windows^9,10^. This results in systematic sequencing of all available peptides only limited by sensitivity. Repeated scanning of the same mass range yields complete elution profiles of both the precursors and the fragments. This increases dynamic range and allows for faster acquisition and deeper proteome characterization down to the single-cell level^11,12^. The principal challenge of DIA is the increased spectral complexity as multiple peptides fragment together leading to convoluted spectra. Thus, DIA data by nature require algorithms to deconvolute overlapping fragmentation patterns and assign peptide identifications.

Initially, DIA involved generating an empirical, sample-specific spectral library, usually acquired by offline fractionation of samples and DDA acquisition or spectrum-centric processing^13,14^. Deconvolution of coisolated peptides into individual spectra effectively reduces them to DDA-like data, amenable to the plethora of proven DDA methods. However, peptide-centric approaches, in which each spectrum of the library is matched to the complex DIA data, achieve higher performance especially if paired with deep-learning-based scoring of identifications as pioneered by Demichev et al.^15–17^. Deep learning also allows the prediction of libraries in silico, obviating the need for sample-specific empirical libraries^18–21^. However, for optimal performance, this has so far required DDA data on the same MS platform and experimental method. This is particularly the case for spectra of post-translationally modified peptides as support for DIA libraries is only emerging^22–24^.

Despite the enormous potential of DIA, the fact that spectra are not easily manually interpretable has hindered full acceptance, especially as researchers must generally rely on a few closed-source algorithms. Flexible and open algorithms would clearly be beneficial to extend the reach, transparency and acceptance of DIA and allow incorporating creative new processing algorithms into existing software frameworks^25–27^. This becomes especially necessary as the most recent generation of instrument uses time-of-flight (TOF) detectors, which are sensitive down to the single-molecule level^28,29^. Raw files easily contain billions of detector events, often with no clearly visible peaks and up to four dimensions of separation^30^. Handling these data has usually required data reduction of the ion mobility dimension, introducing feature boundaries or centroiding^31,32^, which may all lead to loss of information. We found that this presents formidable challenges when implementing novel scan modes that make data processing even more demanding^33–35^.

Therefore, to enable open, performant and extensible processing of high complexity DIA data, we propose a processing framework that builds on current developments in deep learning. Our algorithms view a DIA experiment as a high-dimensional snapshot of the peptide spectrum space. This representation is amenable to DIA methods on all major instrument platforms and naturally covers simple DIA methods, as well as ion mobility, variable windows, sliding quadrupole windows and yet-to-be-developed acquisition modes. Integral to this generalized representation, the data are processed without a reduction in retention time or mobility resolution. Instead, our feature-free approach performs machine learning directly on the raw signal, combing all available information before making discrete identifications. Furthermore, we propose a DIA transfer learning strategy based on our recently published alphaPeptDeep library. Transfer learning adapts the peptide library directly to the instrument and sample workflow^36^. This closer coupling of deep learning beyond library prediction may become characteristic of the next generation of search engines^37^. We showcase performance and versatility by extending DIA to arbitrary peptide PTMs, closing the gap between the versatility of DDA and the performance of DIA.

## Results

**Fig. 1:**
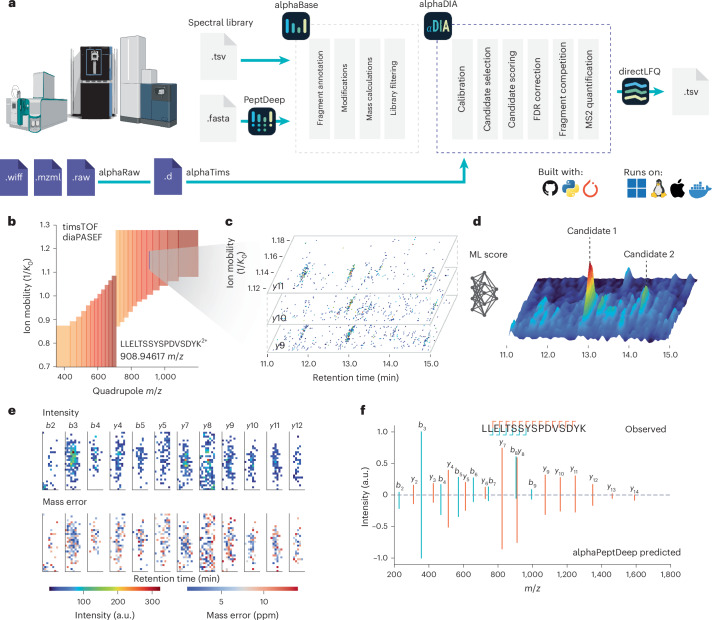
Overview of the alphaDIA framework. **a**, Components of alphaDIA and the integration into the alphaPept ecosystem. AlphaDIA uses alphaRaw and alphaTims^50^ for accessing raw data from all major vendors. Importing and prediction of spectral libraries are facilitated by alphaBase and alphaPeptDeep^20^. After successful search, LFQ is performed using directLFQ^40^. Two leftmost mass spectrometry instrument illustrations created with BioRender. **b**–**f**, TIMS DIA data acquired using optimal dia-PASEF^39^ are searched using a peptide-centric algorithm. **b**, The library entry for a single peptide sequence is selected for search. **c**, Fragment spectra containing the precursor of interest are extracted and converted into a dense matrix in spectrum space. **d**, Information from fragments mapping to the precursor of interest are combined in a continuous score. ML, machine learning. **e**, AlphaDIA defines candidate peak groups with discrete integration boundaries (top row: intensities, bottom row: mass deviation from theoretical mass). **f**, Aggregating signal across the integration boundaries in ion mobility and retention time reveals the peptide spectrum. For further scoring, AlphaPeptDeep spectrum predictions are used.

We present alphaDIA, a modular, open-source framework for DIA search. It builds on the scientific python stack and the alphaPept^38^ ecosystem allowing flexible search strategies and default workflows accessible through a Python API, Jupyter notebooks, a command line interface or an easily installable graphical user interface (Fig. 1a and Methods). AlphaDIA covers the entire workflow from raw files to reporting protein quantities and can process files and proprietary formats from all major vendors. It was designed for ‘one-stop processing’ of large cohorts, running natively on Windows, Linux and Mac or in a distributed fashion in the cloud with Slurm or Docker.

### Feature-free processing for high-dimensional TOF data

**Extended Data Fig. 1:**
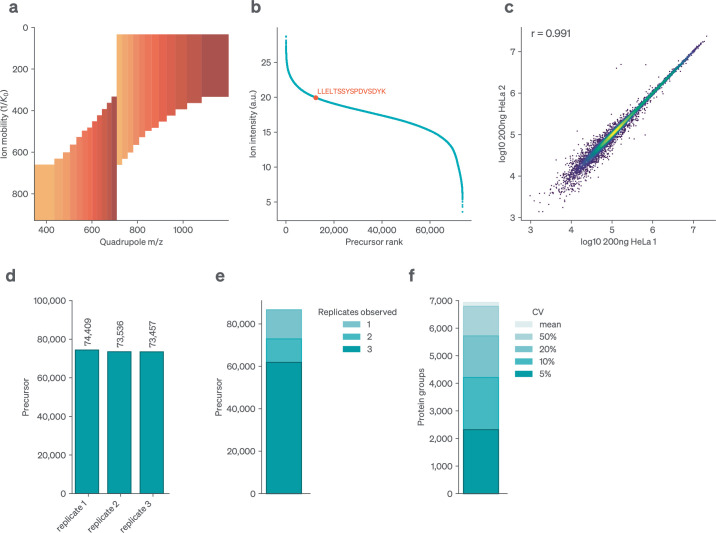
alphaDIA search results for library-based search of triplicate bulk HeLa dia-PASEF data. Data was acquired at 60SPD (21 min) on the timsTOF Ultra. **a**, Overview of the MS2 window distribution scheme of optimal dia-PASEF. **b**, Precursor selected as example in Fig. 1b–f. **c**, Correlation of LFQ protein quantities across replicates. **d**, Number of precursors identified in each replicate at 1% FDR. **e**, Reproducibility of precursor identification across replicates. Number of precursors identified in at least 1, 2 or 3 replicates **f**, Precision of protein quantification. Number of protein groups for given CV cutoffs.

**Extended Data Fig. 2:**
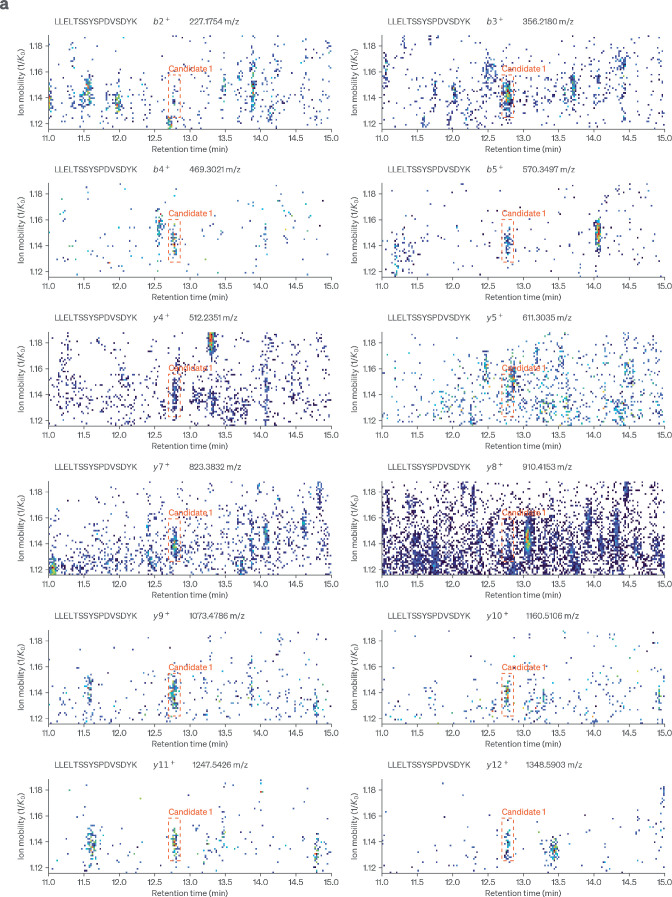
Fragment signal across ion mobility and retention time for the precursor LLELTSSYSPDVSDYK^2+^. **a**, For each fragment all signal within the 15ppm of calibrated mass tolerance is shown as well as the final integration boundaries of the identified precursor. Due to the high sensitivity of time-of-flight detectors fragment signal might only correspond to few ion copies. This leads to stochastic sampling of ions and discontinuous signal across retention time and ion mobility. Distinguishing fragment signal from other ion species is challenging and prevents to determine clear peak boundaries. This requires an algorithm which does not need a minimum number of data points or certain peak shape. It is likewise important to combine evidence across fragments for determination of peak group boundaries.

Apart from state-of-the-art DIA processing, the impetus for alphaDIA was the shift toward fast, sensitive and stochastic TOF detectors, presenting novel algorithmic challenges and opportunities. AlphaDIA’s feature-free and peptide-centric search is illustrated by the identification of the peptide LLELTSSYSPDVSDYK^2+^ from timsTOF Ultra dia-PASEF (parallel accumulation serial fragmentation) data (Extended Data Fig. 1). First, we select all MS1 and MS2 spectra that contribute evidence for this precursor (Fig. 1b). A dense representation of the spectrum space is used to score potential peak group candidates, which does not involve feature building or centroiding (Fig. 1c,d). Instead, signals are aggregated across retention time, ion mobility and fragments using learned convolution kernels. Discrete peak groups are determined only after all this evidence has been collected (Fig. 1e). In this way, noisy TOF data in which individual fragment signals are not distinguishable from background can still be processed (Extended Data Fig. 2). The agreement with the predicted spectrum gives evidence for a confident identification only when the signal in the peak groups is integrated into a spectrum of matched fragments (Fig. 1f).

### Deep-learning-based search for proteome characterization

**Fig. 2:**
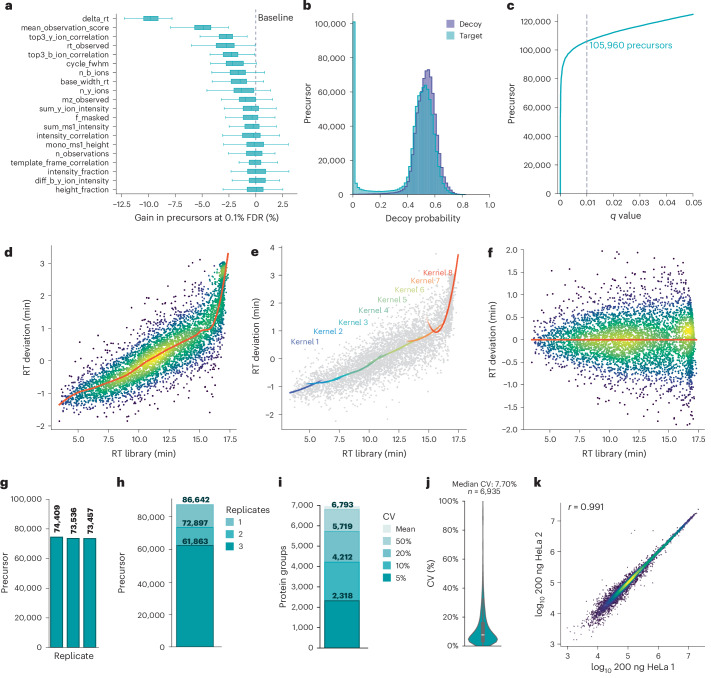
Central search engine components. **a**, Classifier features and their importance for the supervised target–decoy competition. Feature importance is defined as percentage drop of precursor identifications at 0.1% FDR across replicate training with random initial parameters (*n* = 100; box plot defined as per Methods). **b**, Deep NN output probability for decoy peptides. **c**, Number of precursors identified as a function of the *q*-value cutoff. **d**, Nonlinear calibration of retention times using LOESS regression (Supplementary Fig. 1 and Methods). RT, retention time. **e**, Collection of polynomial basis functions combined using local kernels. **f**, Retention time deviation after calibration. **g**–**k**, Results for the library-based search of HeLa lysates measured with dia-PASEF. **g**, Number of precursors identified at a 1% FDR in three replicates. **h**, Precursors shared across replicates. **i**, Protein groups identified at given CVs. **j**, Distribution of protein group CVs (*n* = 3). **k**, Pearson correlation of precursor intensities across samples.

AlphaDIA uses deep-learning-based target–decoy competition and iterative calibration to search complex proteomes with spectral libraries. For each target precursor entry with a given sequence and charge state, a paired decoy peptide is created using a mutation pattern (Methods). Each peak group is scored by a collection of up to 47 features using a fully connected neural network (NN) (Fig. 2a). False precursor identifications are controlled using a count-based false discovery rate (FDR), calculated from the probabilities predicted by the NN (Fig. 2b,c). Measured properties such as retention time, ion mobility and *m*/*z* ratios are iteratively calibrated to the observed data on a high-confidence subset of precursors, using nonlinear locally estimated scatterplot smoothing (LOESS) regression with polynomial basis functions (Fig. 2d–f and Supplementary Fig. 1). AlphaDIA uses spectrum-centric fragment competition to ensure that fragment information is only used for single-precursor identification, even when multiple library entries match the same observed signal (Methods). To assess the performance of this algorithm, we performed a library-based search using a previously published spectral library^39^ from fractionated Hela lysate that was searched with MSFragger. On a 21-min gradient with 60 samples per day (SPD) of HeLa cell lysate measured on a timsTOF Ultra with dia-PASEF, our algorithm identified more than 73,000 precursors with unique sequence and charge, corresponding to almost 6,800 protein groups (Fig. 2g–i). For label-free quantification (LFQ), we integrated the recently developed directLFQ algorithm^40^, which resulted in a median coefficient of variation (CV) of 7.7% for protein groups and a Person *R* > 0.99 across replicates (Fig. 2j,k). This suggests that alphaDIA can search and quantify complex protein mixtures with excellent depth and quantitative precision.

### AlphaDIA adapts to instruments and acquisition methods

**Fig. 3:**
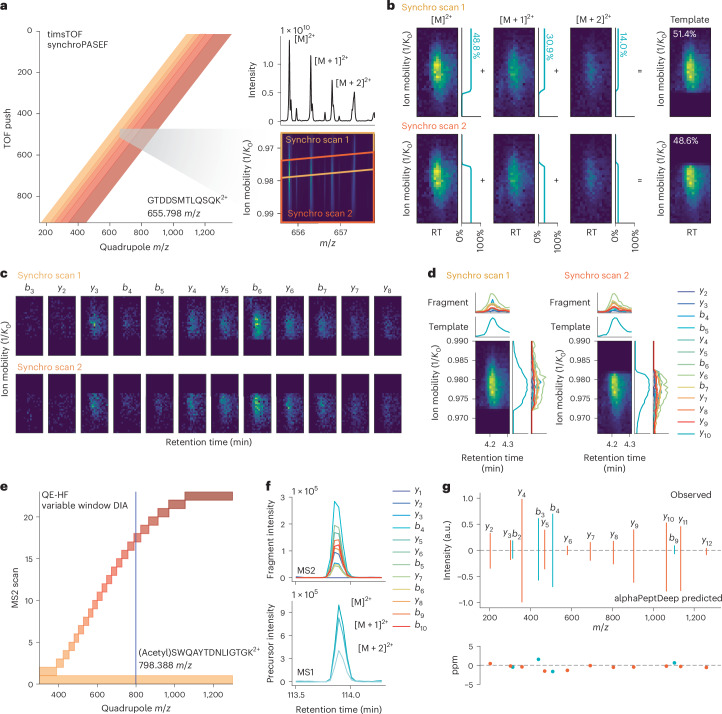
AlphaDIA enables flexible processing for different acquisition methods. **a**, Variable-window synchro-PASEF acquisition on the timsTOF. The precursor with sequence GTDDSMTLQSQK is sliced by the quadrupole, resulting in fragment signal across two synchro scans. **b**, Slicing patterns are resolved by calculating the expected distribution of fragment signal in form of a template matrix. The template matrix is calculated by transforming the individual precursor isotope signal with the quadrupole transmission function of the synchro scans. **c**, Observed fragment signal across the two synchro scans. **d**, For each of the two synchro scans, the elution and ion mobility XICs are compared. Comparison of the fragment signal to the template provides evidence of the identification of peptides. **e**, Application of the processing algorithm to variable-window DIA data without ion mobility separation on a quadrupole Orbitrap analyzer (QE-HF). For the given precursor, all valid MS2 scans contributing evidence are selected. **f**, Elution profile of fragment and precursor ions for the precursor of interest. **g**, Observed and predicted fragment intensities after integration of the peak area (top) and mass accuracy for the same precursor (bottom).

**Extended Data Fig. 3:**
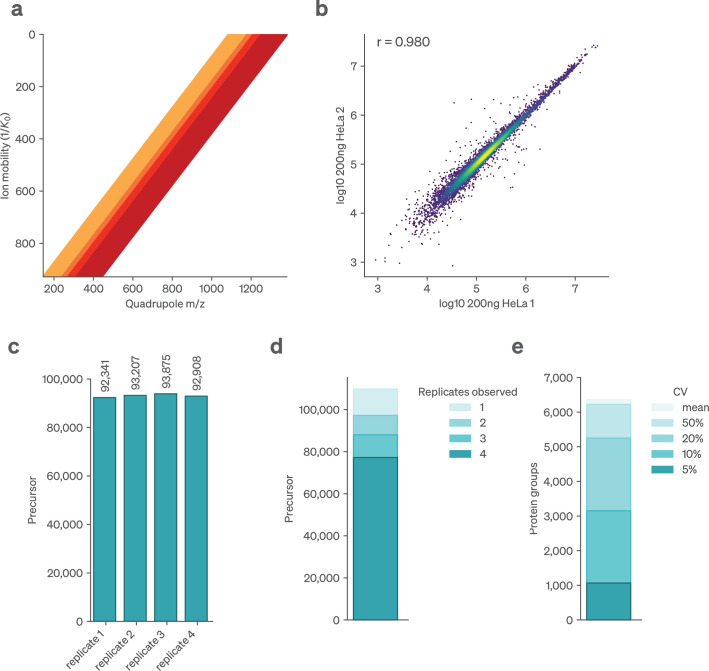
Processing of synchro-PASEF data with alphaDIA. Analysis of bulk HeLa lysate with synchro-PASEF on the timsTOF Ultra. **a**, In synchro-PASEF the quadrupole is continuously scanning across the mass range while ions elute from the TIMS trap. In this method, four synchro scans of variable width are being used. **b**, Correlation of protein groups quantified between two replicates of HeLa lysate **c**, Number of precursors identified at 1% FDR per replicate. **d**, Data completeness given by precursors identified in a minimum number of replicates. **e**, Coefficient of variation (CV) for protein groups.

Recently, DIA has been coupled to sophisticated data acquisition schemes where the quadrupole isolation window scans nearly continuously through the *m*/*z* or *m*/*z* and ion mobility space^11,29,32^. The methods, termed synchro-PASEF or midia-PASEF hold the promise of much improved precursor specificity and quantitative accuracy; however, this has been difficult to realize because of a lack of flexible algorithms handling the thousands of individual isolation windows per DIA cycle. AlphaDIA’s processing algorithm and alphaRaw’s efficient data handling allow using all synchro scans that contribute signal for a given precursor, considering its isotope distribution as a prior (Fig. 3a). Using the masses and abundance of the precursor isotopes, we model the behavior of the quadrupole, resulting in a template with the expected intensity distribution across synchro scan observations (Fig. 3b). This template includes the slicing of the isotope distribution by the quadrupole, which must be recapitulated in the intensity profiles of the fragments (Fig. 3c). This comparison of the fragment profile with the template contributes to our deep-learning-based identification score and enables the analysis of complex proteomes (Fig. 3d and Extended Data Fig. 3). This first processing algorithm for sliding quadrupole data could be extended from synchro-PASEF to similar acquisition schemes such as midia-PASEF or scanning SWATH (sequential window acquisition of all theoretical fragment ions).

**Extended Data Fig. 4:**
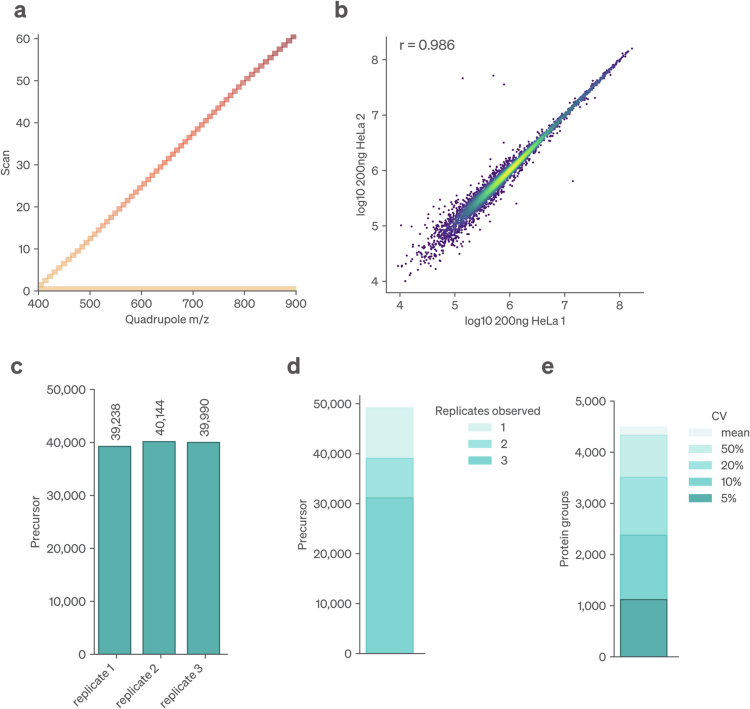
Analysis of Sciex swath data acquired on the ZenoTOF 7600. Bulk HeLa lysate was analyzed with 21 min of active gradient. **a**, Overview of the acquisition method used for data acquisition. The position of MS2 quadrupole windows is shown for a single DIA cycle. **b**, Correlation of protein groups quantified between two replicates of HeLa lysate **c**, Number of precursors identified at 1% FDR per replicate. **d**, Data completeness given by precursors identified in a minimum number of replicates. **e**, Coefficient of variation (CV) for protein groups.

Next, we wanted to extend the reach of alphaDIA to other proteomic platforms and methods. For instance, our algorithms adapted naturally to fixed-window and variable-window DIA data from quadrupole Orbitrap analyzers. The absence of ion mobility reduces the search space to a one-dimensional search across retention time while still using all valid MS2 observations for a given precursor (Fig. 3e). As before, after discrete peak group candidates have been identified (Fig. 3f), the spectrum-centric view allows detailed scoring using alphaPeptDeep-predicted spectra (Fig. 3g). Additionally, alphaDIA can process Orbitrap and Orbitrap Astral data with wide, narrow, variable or overlapping DIA windows. It can likewise process Sciex SWATH data (Extended Data Fig. 4).

### AlphaDIA matches popular packages in library-based search

**Fig. 4:**
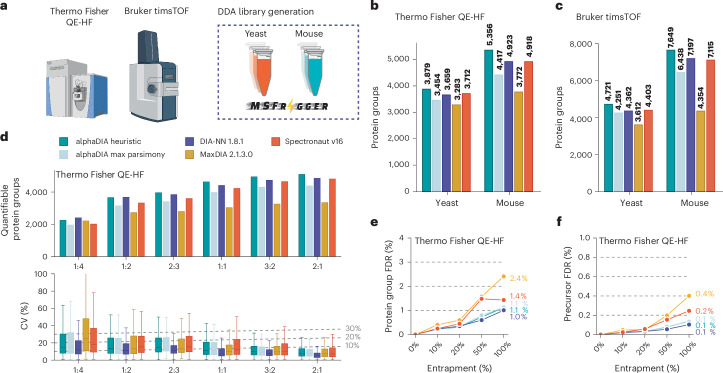
Benchmarking alphaDIA against established software for empirical library-based DIA search. **a**, Overview of the benchmarking dataset^41^ for empirical library-based search acquired on the quadrupole orbitrap QE-HF platform and timsTOF. Fractionated bulk samples were analyzed using DDA to generate sample-specific libraries using MSFragger. Mouse brain membrane isolates were spiked into a complex yeast background at different ratios and analyzed in five replicates using DIA on both platforms. Mass spectrometry instrument illustrations created with BioRender. **b**, Number of Mouse protein groups identified at 1% FDR across all replicates on the QE-HF. **c**, Same as **b** but on the timsTOF platform. **d**, Quantified mouse protein groups between different spike-ins and a reference sample across five replicates. The CV is shown for each set of identifications (box plot defined as per Methods). **e**, Benchmarking of FDR using increasing numbers of *Arabidopsis* entrapments compared to the yeast/mouse spectral library. The FDR on the protein level is shown for the QE-HF platform. **f**, Same as **e** but on the precursor level.

Having established the ability of alphaDIA for in-depth analysis of complex proteomes and its adaptability to diverse platforms, we next wanted to directly benchmark its performance against other common DIA search engines. To avoid potential bias, we build upon a recently published benchmarking study from the Shui group, in which mouse brain membrane isolates were spiked into a complex background of yeast proteins in varying ratios and measured on a quadrupole orbitrap (QE-HF) and a timsTOF^41^. The authors generated empirical libraries with MS Fragger^4^ and optimized search parameters for DIA-NN, Spectronaut and MaxDIA (Fig. 4a).

**Extended Data Fig. 5:**
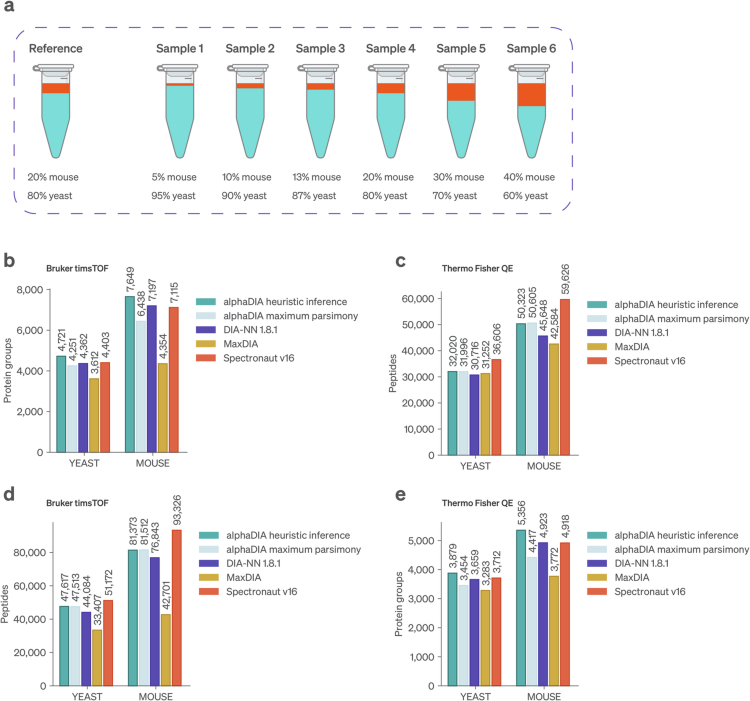
Benchmarking library based search in a complex background. **a**, Experimental setup as described by Lou et al.^33^ Mouse brain isolate digests were spiked into a complex yeast proteome background in different ratios and measured in five technical replicates. **b**, Protein groups identified at 1% FDR on the Bruker timsTOF. **c**, Protein groups identified at 1% FDR on the Thermo Fisher QE-HF. **d**, Unique modified peptides identified 1% FDR across replicates on the Bruker timsTOF. **e**, Unique modified peptides identified 1% FDR across replicates on the Thermo Fisher QE-HF.

On the basis of the provided libraries, alphaDIA identified up to 50,600 mouse peptides in the QE data across all samples and up to 81,500 on the timsTOF (Extended Data Fig. 5). Inferring proteins from uniquely identified peptide involves considerations that can influence the number of reported protein groups^42^. AlphaDIA allows strict (maximum parsimony) or commonly used heuristic grouping (Methods). With the latter, we identified 5,366 proteins (QE-HF) and 7,649 (timsTOF) protein groups across all samples, matching and even exceeding the other algorithms (Fig. 4b,c). This is also reflected across replicates for single conditions. AlphaDIA quantified the most protein groups in at least three of five replicates for most ratios while maintaining comparable CVs and accuracy as judged by the proteome mixing ratios (Fig. 4d and Supplementary Figs. 2 and 3).

To prevent over-reporting by sophisticated DIA database searching strategies based on internal target–decoy FDR estimates, results can be externally validated by including additional proteome databases from species not present in the sample^43^. As in the benchmarking study, we performed an entrapment search with an *Arabidopsis* library added in increasing proportions to the target library. On both MS platforms, even for 100% entrapment, *Arabidopsis* identifications matched the chosen target FDR of 1% at the protein level (Fig. 4e,f). At this protein FDR, false-positive precursors are even less likely, appearing only at 0.1% globally. This contrasted with some of the other tested tools, which reported up to threefold more false-positive *Arabidopsis* identifications than intended at the chosen FDR target (Supplementary Fig. 4). The increased library size only minimally decreased overall identifications for alphaDIA. We conclude that, for library-based search, alphaDIA provides at least competitive performance with common search engines while maintaining a reliable and conservative FDR.

### Predicted library search with alphaPeptDeep

While empirical libraries benefit from implicitly capturing instrument and workflow specific properties, the key advantage of deep-learning-predicted libraries of the entire proteome database is that it eliminates cumbersome library measurement altogether. We recently introduced alphaPeptDeep, an open-source, transformer-based deep learning framework for predicting all MS-relevant peptide properties from their sequences^20^.

**Fig. 5:**
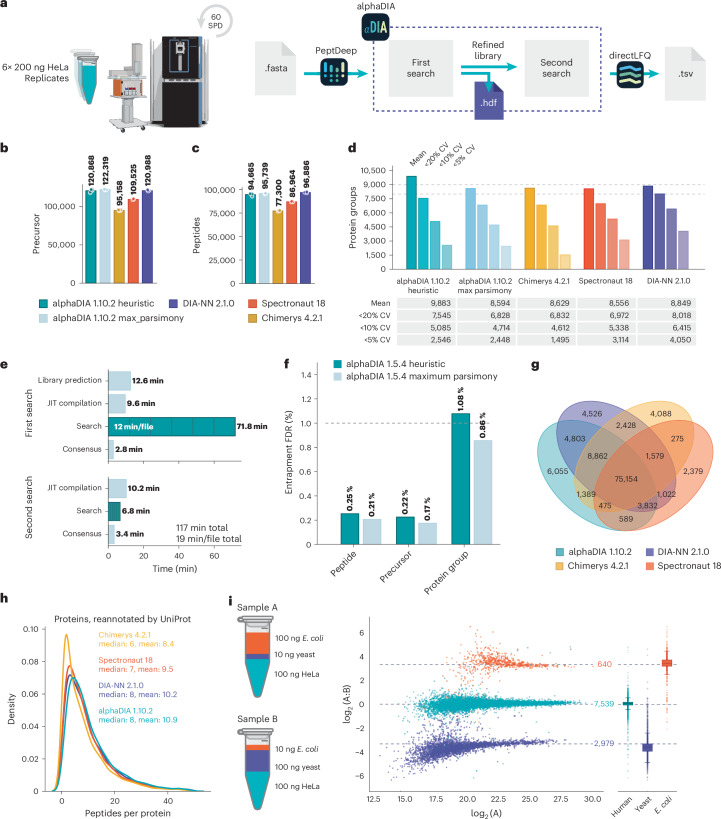
Searching complex proteomes acquired on the Orbitrap Astral with fully predicted spectral libraries. **a**, Six replicates of 200-ng HeLa bulk data were analyzed on the Orbitrap Astral with a 60-SPD (21 min) gradient. A fully predicted alphaPeptDeep library was used for a two-step search in alphaDIA. Different search engines were used for comparison. Evosep liquid chromatography illustration created with BioRender. **b**, Mean precursors identified across search engines (*n* = 6) **c**, Mean modified peptides identified across processing methods (*n* = 6) **d**, Protein groups identified at given CV cutoffs. **e**, Analysis time for different processing steps when analyzed with on a 32-core machine. **f**, *Arabidopsis* entrapment search using the fully predicted library workflow. The share of identified *Arabidopsis* proteins at 1% target–decoy FDR is shown. **g**, Venn diagram showing the overlap of proteotypic peptides across processing methods. **h**, Analysis of protein overlap between processing methods. Peptides were mapped back to the same reference proteome, discarding ambiguous matches. The median number of peptides per protein is shown. **i**, Mixed-species experiment for establishing quantitative accuracy. Human, yeast and *E.* *coli* proteomes were combined in defined ratios and protein ratios are shown for proteins quantified in at least three of five replicates (box plot defined as per Methods).

**Extended Data Fig. 6:**
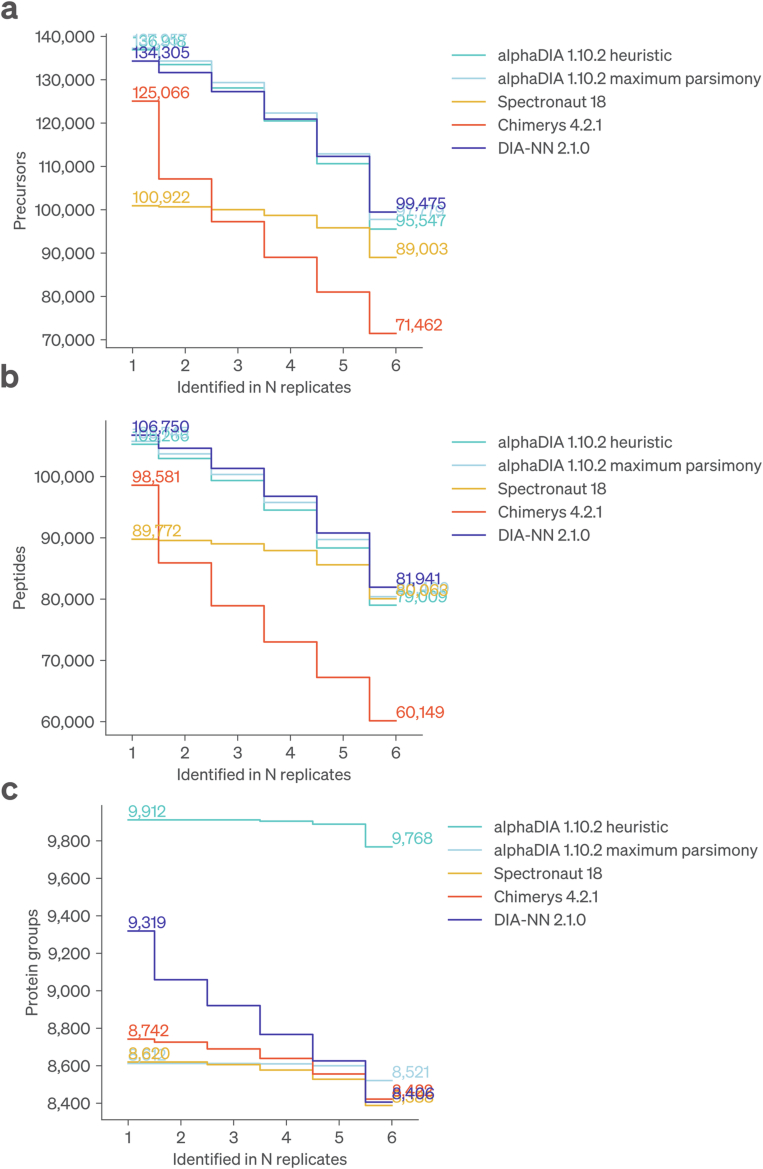
Comparison of data completeness across search engines. **a**, Data completeness of precursor identifications across replicates. **b**, Data completeness of modified peptide identifications across replicates. **c**, Data completeness of protein identifications across runs.

With these state-of-the-art predicted libraries, we devised a two-step search workflow in alphaDIA consisting of library refinement and quantification (Fig. 5a). Furthermore, we reasoned that our feature-free search should adapt well to the high-sensitivity TOF data generated by the Orbitrap Astral MS instrument. For benchmarking, we acquired and searched bulk Hela samples with an alphaPeptDeep-predicted library containing 3.6 million tryptic precursors. AlphaDIA identified on average more than 120,000 precursors, matching or exceeding the performance of the other tested search engines (Fig. 5b). As comparison of inferred protein numbers in bottom-up proteomics depends on the chosen algorithm, which is not public for the other tools, we wanted to provide an upper and lower limit with heuristic grouping and more conservative maximum-parsimony-based inference (Methods). Remarkably, in the 60-SPD method (21 min) this corresponded to the identification of 9,800 protein groups with heuristic grouping and close to 8,600 proteins without grouping (Fig. 5d). The great depth of proteome characterization was also reflected in the data completeness across replicates (Extended Data Fig. 6). We validated the FDR control of this more complex two-step workflow by appending the *Arabidopsis* library, which externally confirmed rigorous control of false-positive identifications (1.08% at protein level and 0.2% at precursor level; Fig. 5f). While searches of fully predicted tryptic libraries are usually faster than acquisition for non-ion-mobility data (Fig. 5e), the explicit modeling of the ion mobility dimension leads to increased processing times (>1 h per file) for large libraries and will need improvement in future versions of AlphaDIA.

**Extended Data Fig. 7:**
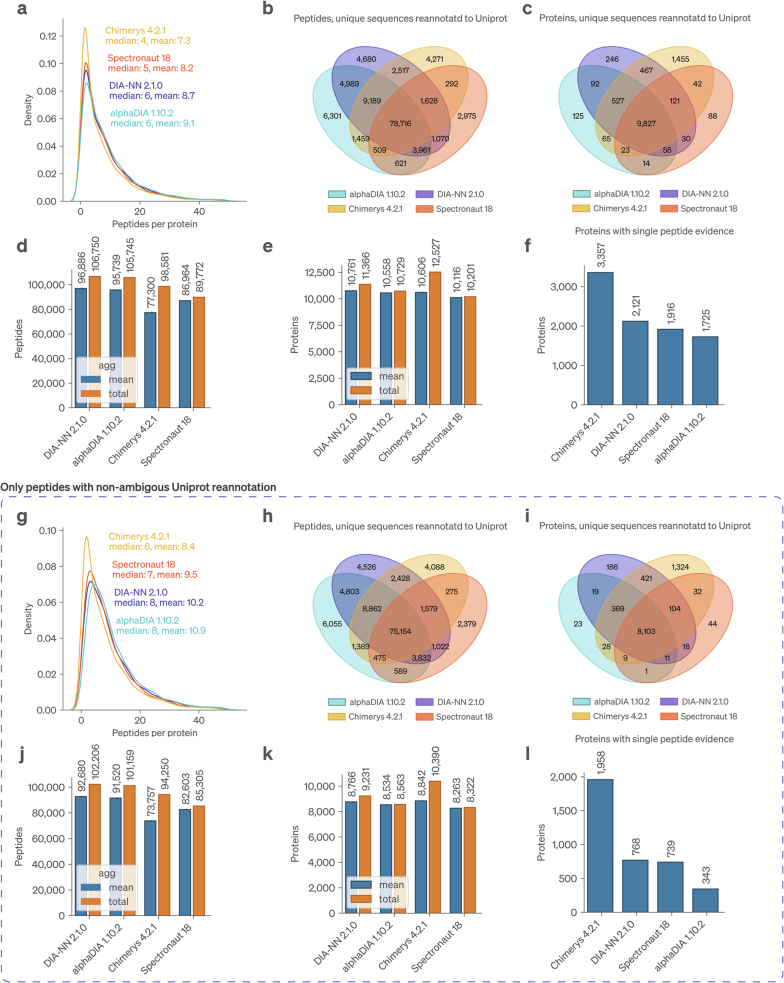
Comparison of peptide and protein identification after remapping to Uniprot. To compare protein search performance independent of protein grouping effects, search engine results were mapped back to the human Uniprot reference proteome. Peptide and protein level identifications were compared for all peptides (**a**-**f**) as well as for only non-ambiguous peptide matches (**g**-**l**). **a**,**g**, Distribution of peptides per protein across all searched samples for. **b**,**h**, Venn diagram comparing unique peptides identified by the different search engines. **c**, Venn diagram comparing protein groups identified by different search engines, without performing protein inference. **i**, Venn diagram comparing proteins identified by different search engines with protein specific evidence. **d**,**j** Number of peptides identified at 1% FDR on average per sample (mean) and across all samples (total). **e**,**k**, Number of proteins identified at 1% FDR on average per sample (mean) and across all samples (total). **f**,**l**, Proteins identified at 1% FDR backed by only a single peptide sequence across all samples.

To compare identified proteins across search engines, we mapped peptide sequences to the UniProt reference proteome. Reassuringly, more than 78,000 peptides and 8,100 proteins (counting only nonambiguous matches) were jointly identified by all tested tools (Fig. 5g). AlphaDIA had the highest number of uniquely identified peptides among search engines, manifesting in high sequence coverage (median of eight peptides per protein; Fig. 5h) and few proteins with only single-peptide evidence across the tested search engines (Extended Data Fig. 7).

**Extended Data Fig. 8:**
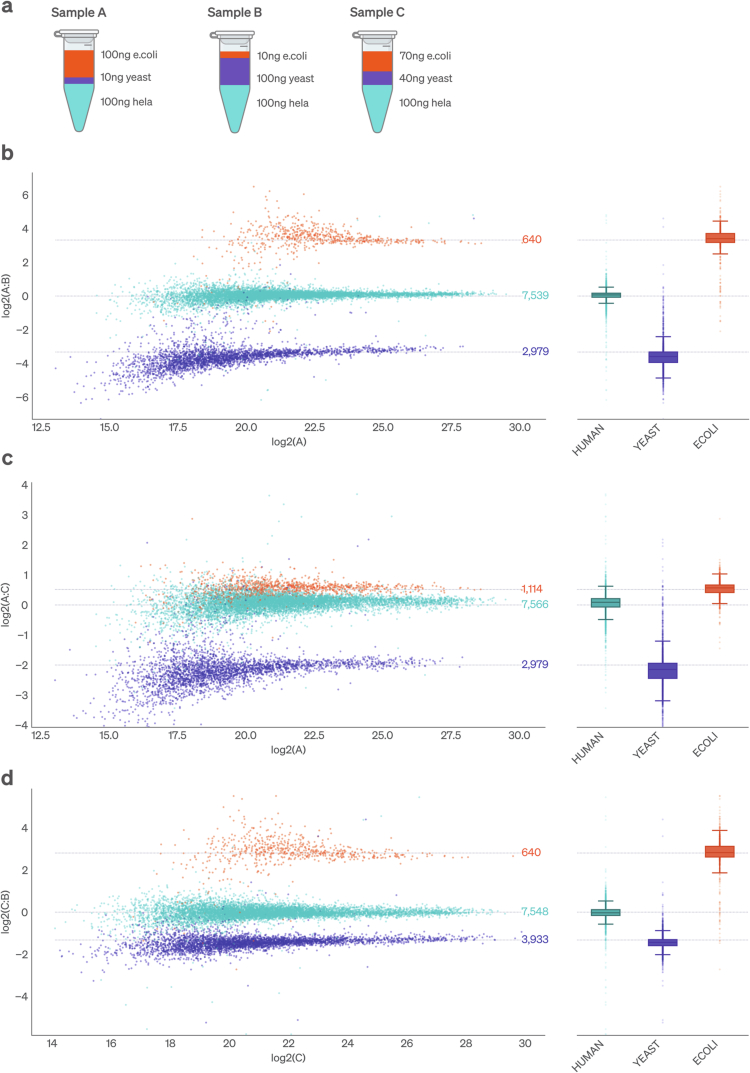
Quantitative accuracy benchmark using mixed species proteomes on the Orbitrap Astral. **a**, Five replicates of three samples were prepared with Yeast, E.coli and human proteomes mixed in defined ratios. Ratios are shown for proteins quantified in at least three out of five replicates. (boxplot defined as per Methods) **b**, Comparison of median protein group intensities at 1% FDR between sample A and B. **c**, Comparison of median protein group intensities at 1% FDR between sample A and B. **d**, Comparison of median protein group intensities at 1% FDR between sample C and B.

To assess the accuracy of LFQ, we used the established strategy^44^ of three species proteomes mixed in defined ratios, acquired on the Orbitrap Astral. Fully predicted library search combined with directLFQ recapitulated the expected ratios with excellent precision and accuracy (Fig. 5i and Extended Data Fig. 8).

**Extended Data Fig. 9:**
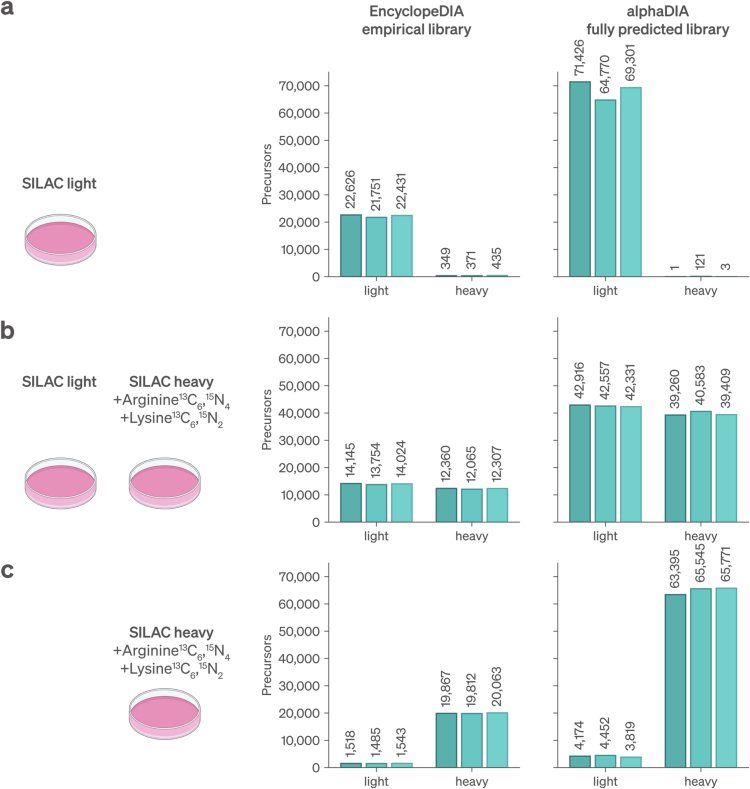
Validation of identification in SILAC labeled samples. SILAC data is from a method optimization study by the Garcia group that was originally analyzed by EncyclopeDIA and an empirical library^50^. This is compared to a fully alphaPeptDeep predicted library and database search by AlphaDIA. Triplicates results from the original paper are plotted in the left-hand panels and the AlphaDIA results on the same data in the right-hand panels. **a**, Percentage of false identifications in the heavy channel are median of 1.6% with EncyclopeDIA and 0.0043% with alphaDIA, which identified a threefold more precursors. **b**, For the combined sample, the heavy to light ratios are similar (46.7% heavy in EncyclopeDIA to 48.1% heavy in alphaDIA). **c**, After extended incorporation both analyses found similar percentage of light peptides (7.1% light in EncyclopeDIA vs 6.0% light in alphaDIA).

Multiplexed DIA has recently shown great potential to increase throughput and depth^45,46^. To analyze such data, identifications must be transferred between the channels, which involves an additional channel FDR. We benchmarked it to a DIA dataset in which HeLa cells were labeled as heavy and light using stable isotope labeling by amino acids in cell culture (SILAC) and analyzed on a QE-HFX^47^ (Extended Data Fig. 9). Proportions of identifications in ‘light only’, ‘heavy only’ and ‘light and heavy’ were very similar to the previous DDA and DIA results, validating our channel FDR. Interestingly, on the same data, the absolute number of identified peptides was threefold higher than in the original paper, reflecting advances in DIA search over the last years in general and specifically in alphaDIA.

### DIA transfer learning allows search with unseen PTMs

**Fig. 6:**
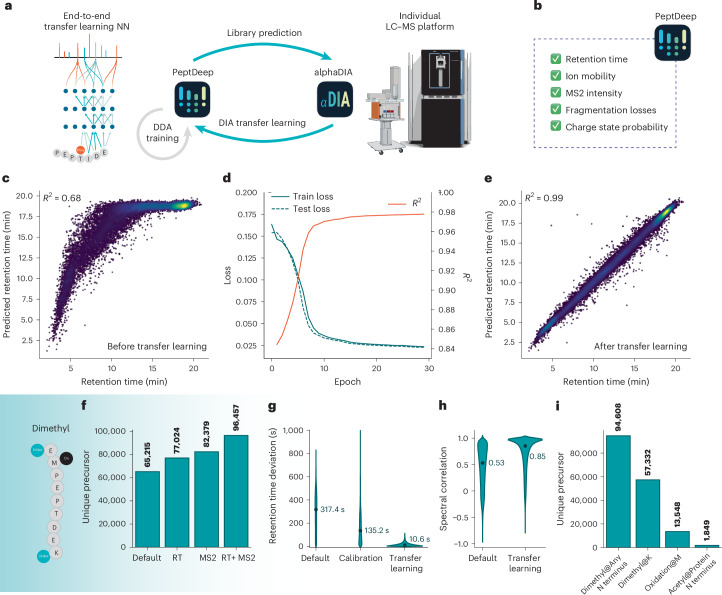
DIA transfer learning for discovery of modified peptides. **a**, A custom deep learning model was trained for every experiment using the identifications from the DIA search engine. Evosep liquid chromatography illustration created with BioRender. **b**, Multiple properties were optimized, resulting in smaller and better matching spectral libraries. **c**, Observed and predicted retention times for dimethylated precursors before transfer learning. **d**, DIA transfer learning for the retention times of dimethylated peptides. During training by stochastic gradient descent, a 20% validation set of precursors was held out to mitigate overfitting and ensure generalization to the peptide space of interest. **e**, Retention times after transfer learning. **f**, Comparison of the number of unique peptides identified with the pretrained base model (default) to the transfer learned model after retention time and MS2 transfer learning. **g**, Distribution of absolute retention time errors for the pretrained base model (default), the nonlinear calibration within alphaDIA and after transfer learning. **h**, Comparison of spectral correlation before and after MS2 transfer learning. **i**, Number of unique observed modifications by type.

To date, fully predicted libraries address many of the needs of DIA workflows but their pretrained prediction models are still best suited to the sample and instrument types that were used in training. This makes it necessary to train custom models for different situations (for example, PTMs), as they generally change retention and fragmentation behavior compared to the unmodified peptide. We reasoned that close integration of prediction by deep learning and the search engine might have the potential learn to adapt to such differences, an approach that we call DIA transfer learning. The subsequent search with alphaDIA confidently identified precursors and their spectra were first collected into a training dataset. The general pretrained models for retention time, fragmentation spectra and charge were fine-tuned on the experiment-specific training dataset (Fig. 6a,b). This resulted in a custom model, reflecting the behavior of peptides on the individual LC–MS setup. A hold-out validation and test dataset ensured generalization and prevented overfitting.

To assess the potential of transfer learning, we first applied it to a dataset of dimethylated HeLa peptides, an example of a modification that is known to alter retention times and fragmentation behavior (Methods and Fig. 6c). We found that transfer learning accurately modeled the effects of the lysine and N-terminal dimethylation on retention time behavior, improving *R*^2^ from 0.69 to 0.99 (Fig. 6d–i).

Using the transfer learned model resulted in a total of 96,000 unique precursor and 8,613 protein identifications, a 48% increase over the 65,000 precursors identified without transfer learning and a 25% increase in protein groups (Fig. 6d,e and Supplementary Fig. 5). This gain in identifications is driven additively by both improved predictions of retention times from a median prediction error of 317 s down to only 11 s and an increase in the median correlation to predicted spectra from 0.5 to 0.85 (Fig. 6g,h).

**Extended Data Fig. 10:**
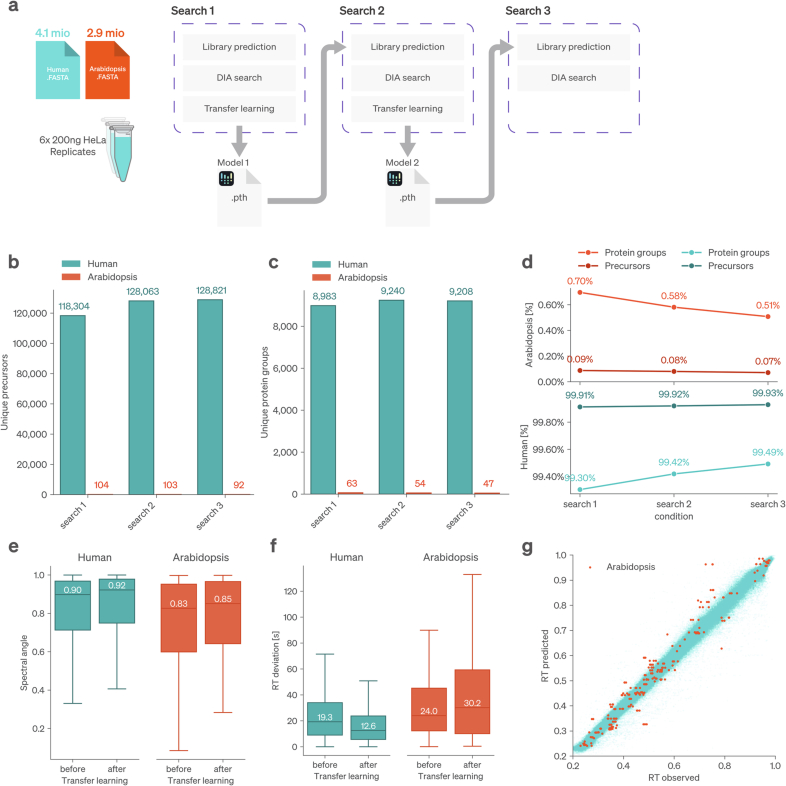
Entrapment validation of end-to-end transfer learning across four iterations. **a**, Overview of the validation workflow. A Human and Arabidopsis fasta file digest was used for fully predicted library search. All identified precursors at 1% FDR were subsequently used for DIA transfer learning, including false positive Arabidopsis identifications. This process was repeated twice, using the transfer learned deep-learning model for library prediction. **b**, Total unique identified precursors across six replicates. Precursors mapping to both species, including leucine and isoleucine pairs were removed. **c**, Total unique identified protein groups. **d**, Entrapment FDR given as the percentage of false positive Arabidopsis identifications. **e**, MS2 spectral angle for precursors before and after transfer learning. Median spectral angle is shown for each plot (n_Human_=283,383, n_Arabidopsis_ = 234, boxplot according to Methods). **f**, Retention time deviation in seconds before and after transfer learning. The median retention time deviation is shown across three replicates (n_Human_=283,383, n_Arabidopsis_ = 234, boxplot according to Methods). **g**, Predicted vs observed retention time following transfer learning. False positive Arabidopsis identifications are highlighted.

Given these large improvements, we wished to ascertain that they were not the result of overfitting, despite the use of a hold-out validation and test dataset. Similarly to before, we used entrapment with the *Arabidopsis* proteome library followed by transfer learning with all precursors, including false-positive *Arabidopsis* hits (Extended Data Fig. 10a). Remarkably, even successive rounds of transfer learning led to more confident precursors identifications and <0.5% false *Arabidopsis* identifications at 1% FDR (Extended Data Fig. 10b–d). Upon inspection, we found that predictions of target hits showed substantial improved agreement with observed data, whereas the opposite was true of false-positive *Arabidopsis* hits (Extended Data Fig. 10e–g). This implies that end-to-end transfer learning generalizes to the peptide behavior in the actual experiment, improving identifications and control of false discoveries at the same time.

## Discussion

AlphaDIA addresses critical DIA challenges including spectral data complexity and the need for robust algorithms handling high-dimensional data.

Our results demonstrate that already the first public version of alphaDIA matches and, in many cases, surpasses existing software tools in terms of performance and versatility.

AlphaDIA’s feature-free processing method is central to its performance and flexibility. Traditional DIA processing methods rely on predefined feature boundaries, which can lead to information loss, especially with the high sensitivity and the stochastic nature of TOF detectors. By contrast, alphaDIA’s approach aggregates signals across multiple dimensions, ensuring that all relevant data are used before making discrete identifications. Additionally, alphaDIA extends the reach of DIA to novel acquisition modes. Together with its open-source architecture, alphaDIA enables the community to quickly iterate between experimental innovations and their algorithmic implementation.

Our benchmarking against established tools using both empirical and predicted libraries showcases alphaDIA’s equal or superior performance. This holds true across platforms and experimental designs including the Orbitrap Astral, where alphaDIA identified over 120,000 precursors and 9,800 protein groups in a 60-SPD format.

One of the most innovative aspects of alphaDIA is its transfer learning capability. Through integration with the transformer models of alphaPeptDeep, alphaDIA closes the loop between spectral library prediction and DIA search. Our approach allows the model to adapt to experiment-specific conditions, enhancing the accuracy of peptide identifications. We showcased this on a dataset of dimethylated HeLa peptides demonstrating substantial improvements in retention time prediction and spectral correlation, resulting in a 48% increase in unique precursor identifications and a 25% increase in protein groups compared to using pretrained models alone. This allows the application of DIA search to hitherto inaccessible areas such as post-translationally modified proteins without PTM specific pretraining or to the better identification of HLA peptides. We demonstrated that transfer learning not only improves overall identifications but even improves FDR control, ensuring reliable results. The value of this approach is further validated by the independent and parallel development of Carafe, highlighting a convergence in the field toward transfer learning as a standard tool in DIA processing^48^.

The advancements presented by alphaDIA pave the way for more comprehensive and accurate proteomic analyses, which will be important as MS technology continues to evolve. The framework’s open-source nature ensures that it can be continuously improved and extended by the scientific community, fostering innovation and collaboration^49^. We, therefore, aim to establish alphaDIA as a cornerstone for the next generation of DIA analysis, closely coupled to the developments in artificial intelligence.

## Methods

### Calibration and optimization of retention time, ion mobility and *m*/*z*

During search, AlphaDIA calibrates library properties such as retention time, ion mobility, precursor *m*/*z*, fragment *m*/*z* and search tolerances. Calibration removes the systematic deviation of observed and library values. Optimization reduces the search space to improve the confidence in identifications and to accelerate the search. Initial parameters are an MS1 tolerance of 30 ppm, MS2 tolerance of 30 ppm, 0.1 1/*K*_0_ ion mobility tolerance and 50% retention time tolerance.

AlphaDIA supports search space optimization with fixed target values such as a mass tolerance of 7 ppm and automatic optimization to give optimal search results. By default, mass tolerances are optimized with targeted optimization and retention time while ion mobility tolerances undergo automatic optimization. First, all targeted optimizations are performed at the same time, followed by separate automated optimizations of the remaining properties.

For each optimization, the search is performed batch-wise, starting with the first 8,000 precursors and using an exponential batch plan (16,000, 32,000, 64,000, …) until 200 precursors are identified at 1% FDR. For targeted optimization, the search space of the property of interest is updated to the 95% percentile of the precursors identified at 1% FDR. For automated optimization, the search space of the property of interest is set to the 99% percentile of the precursors identified at 1% FDR and a figure of merit is logged. MS1 error optimization uses the correlation of the observed and predicted isotope intensity profile as a figure of merit. For MS2, retention time and ion mobility use the precursor proportion of the library detected at 1% FDR as a figure of merit. Optimization is stopped if the property of interest does not change substantially. The optimal value based on the figure of merit is used.

Calibration of systematic deviations happens in parallel on the basis of the subset of confident precursors identified at 1% FDR. Library-encoded values are calibrated to match the dataset distribution using LOESS regression. For calibration of fragment *m*/*z* values, up to 5,000 (but at least 500) of the best fragments according to their extracted ion chromatogram (XIC) correlation are used.

LOESS regression with uniformly distributed kernels is used for each property to be calibrated (Supplementary Fig. 1). Regression is performed on first-degree and second-degree polynomial basis functions of the calibratable property. For *m*/*z* and ion mobility, two local estimators with tricubic kernels are used. For retention time prediction, six estimators with tricubic kernels are used. The architecture is built on the scikit-learn package and can be configured to use different hyperparameters and arbitrary predictors for calibration.

### Scoring of precursors and decoys using convolution kernels and supervised classification

AlphaDIA uses a two-step scoring machine learning algorithm to identify the best potential peak group for every library entry. The first step builds on a collection of weighted convolution kernels, learned during optimization and calibration of the spectral library. For every precursor of interest, MS1 scans and MS2 scans contributing information toward the identification are identified from the DIA cycle pattern of the acquisition method. On the basis of a certain number of highest-intensity fragments in the library (default: 12), dense representations of the search space in ion mobility and retention time dimension are assembled. To identify putative peak groups for each precursor, a set of convolution kernels, reflecting the expected distribution in retention time, ion mobility and fragment intensity, are learned during calibration and optimization. The convolution of the search space is performed in Fourier space for fast processing and a single score is calculated as a log sum across kernels and fragments. Local maxima are identified using a simple peak-picking algorithm and retention time and ion mobility boundaries of the peak group of interest are defined from the joint scoring function. These candidates are subsequently rescored for FDR estimation.

As the second step, AlphaDIA uses target–decoy competition for scoring the quality of precursor spectrum matches. Upon library import, paired known false-positive decoy peptides are created for every target. By default, a mutation pattern GAVLIFMPWSCTYHKRQENDBJOUXZ>LLLVVLLLLTSSSSLLNDQEVVVVVV is used. For every library entry, target and decoy, the best high-scoring matches from the convolution kernel score are used for supervised classification. Up to 47 features are calculated for each peak group match, reflecting the merit of the identification. A multilayer perceptron (MLP) deep NN with layer sizes of 100, 50, 20 and 5 and a total of 47 input dimensions (10,810 parameters) is trained to predict the probability of being a false decoy identification. Training is performed with stochastic gradient descent for ten epochs with a batch size of 5,000 and learning rate of 0.001. While training on an 80% training set, a 20% test set is held out to mitigate overfitting. On the basis of the final score, the best (lowest) decoy probability peak group is retained for every library entry and a count-based FDR is calculated.

### FDR calculation

AlphaDIA uses a count-based FDR on the level for assigning confidence to precursor, peptide, protein and channels. Identifications are given as a set of target and decoy identifications $P=\{{p}_{0},{p}_{1},\ldots, {p}_{i}\}$, all associated with a ground-truth decoy status ${\rm{decoy}}:P\to \{{\rm{true}},{\rm{false}}\}$ and a deep-learning-derived decoy score $\hat{y}:P{\mathbb{\to }}{\mathbb{R}}$. For every precursor with index *i*, the number of targets with lower or equal decoy probability,

$${n}_{\rm{target}}=|\{p\,|\;\hat{y}\left(p\right)\le \hat{y}\left({p}_{i}\right),{\rm{decoy}}\left(p\right)={\rm{false}}\}|,$$

and the number of decoys with lower or equal decoy probability,

$${n}_{\rm{decoy}}=|\{{p|}\;\hat{y}\left(p\right)\le \hat{y}\left({p}_{i}\right),{\rm{decoy}}\left(p\right)={\rm{true}}\}|,$$

are calculated. Furthermore, the total numbers of targets and decoys in the set are calculated as follows:

$${N}_{\rm{target}}=|\{{p|{\rm{decoy}}}\left(p\right)={\rm{false}}\}|$$

$${N}_{\rm{decoy}}=|\{{p|{\rm{decoy}}}\left(p\right)={\rm{true}}\}|$$

The local count-based *q* value is given as follows:

$${q}_{i}=\frac{{n}_{\rm{decoy}}}{{n}_{\rm{target}}}\times \frac{{N}_{\rm{target}}}{{N}_{\rm{decoy}}}$$

This is converted to the FDR using the minimum *q* value where a precursor was accepted:

$${\rm{FDR}}_{i}=\min \left({q}_{i},\{q,|,\hat{y}\left(p\right) > \hat{y}\left(\;{p}_{i}\right)\}\right)$$

By default, all identifications are filtered on a run-level 1% FDR precursor threshold and global 1% protein group-level threshold.

### Spectrum-centric fragment competition

Competition of precursors for a fragment ion is used as a spectrum-centric element to mitigate double use of fragments for multiple identifications from the same spectra. Following initial FDR calculation, precursor candidates are filtered at 5% FDR and split into groups of potentially fragment sharing. This is determined by the quadrupole cycle pattern. Then, precursor candidates and their elution width at half maximum are compared so that precursors with overlapping elution width at half maximum have no more than ${k}_{\max }=1$ shared fragment masses within the chosen MS2 mass accuracy ${\delta }_{\rm{MS2}}$. If two or more precursor candidates share more fragments than permitted, the precursor candidate with the lowest decoy score is used.

### Protein inference

Reporting all proteins whose sequence can be matched to any identified peptide can lead to inflation of false discoveries on the protein level^51^. Following the approach outlined by Nesvizhskii et al.^52^, we consider a precursor as a single piece of evidence and the task of protein inference is then to assemble these precursors into proteins while controlling the accumulation of spurious protein identifications. AlphaDIA aims to implement a simple and transparent inference approach, allowing for three inference modes: library, maximum parsimony and heuristic. Apart from the library mode, which uses the inference performed during empirical library creation, protein inference is based on an implementation of the ‘greedy set cover’ algorithm with grouping by default (heuristic) and without grouping for strict inference (maximum parsimony).

In brief, alphaDIA’s protein inference starts with a table of identified precursors. Each precursor is associated with a set of genes and proteins and based on user choice, the inference is performed on the gene or protein level (default: gene). While a common peptide precursor may match many proteins, a proteotypic peptide will match one single protein. During grouping, the precursor and protein arrays are reshaped into a protein-centric view, where each protein is associated with one set of precursors. Then, proteins are sorted by the length of their precursor set in descending order, and the protein with the largest number of precursors removed from the lists as the first query. The query is compared to all remaining subject proteins. From each subject precursor set, all precursors matching the query set are removed. If a protein’s precursor set becomes empty, it is considered redundant and dropped. After all precursor sets have been compared, the process repeats by reordering the list and extracting the next query. After completion, retained queries are denoted master proteins, necessary to explain all discovered precursors. In strict maximum parsimony mode all master proteins are simply reshaped to precursor-centric format, linking each precursor to one single protein ID. In the heuristic mode, the list of master proteins is used to remove all non-master proteins from the initial precursor table, effectively leaving each precursor with a set of associated proteins comprised solely of master proteins. Thereby, the same precursor can be claimed by different proteins, creating protein groups (see also the tutorial notebook in the GitHub repository).

### Protein FDR

Protein FDR is performed on the protein groups calculated during protein inference. For all target and decoy protein groups, seven features are calculated: the total number of precursors across runs for the protein group, the mean decoy score for precursors across runs for the protein group, the number of unique peptides for the protein group, the number of unique precursors for the protein group, the number of runs the protein group was found in, the lowest decoy score across precursors for the protein group and the highest decoy score across precursors for the protein group. We use an MLP to classify decoy protein groups from target protein groups. Correct training is ensured by a 20% held-out test set. Protein group FDRs are calculated on a global level using the FDR mechanism described above.

### Library refinement for fully predicted libraries

AlphaDIA uses an established two-step search strategy for library refinement^15^. Following an initial search of all or a subset of raw files, protein inference and FDR are determined as configured by the user. All precursors are automatically filtered at 1% local precursor FDR and global 1% protein group FDR, accumulated into a spectral library and finally saved to the project folder. For each precursor, the identification with the best (lowest) decoy probability is used. By default, MS2 quantities are used as annotated in the original library. If transfer learning accumulation is used, custom user specified fragment types can be selected and observed MS2 intensities are extracted. This spectral library is then used for the second search with full MS2-based target–decoy scoring without any relaxed FDR parameters. For protein inference and FDR, library-annotated protein groups are used.

### Transfer learning

To create transfer learning libraries, precursors identified at 1% precursor and protein FDR are selected for requantification. Precursors are requantified for user-defined fragment ion types (*a*, *b*, *c*, *x*, *y*, *z*, modification loss, etc.) and a user-defined maximum charge (default: 2). Extracted fragment quantities are accumulated across samples and ordered by their decoy probability. For each unique modified precursor, the observations with the three lowest decoy scores are selected. AlphaDIA also creates a high-quality subset where only precursors with a median fragment correlation greater than 0.5 are included. For these precursors, we only retain fragments whose correlation values exceed 75% of the median fragment correlation of the respective precursor. The implementation of transfer learning library is globally sequential. At any given time, we can limit the implementation to only parallelize across a limited number of processes. This approach allows the process to scale without storing all runs in memory.

For transfer learning, we prioritized robustness to ensure performance instead of requiring users to define hyperparameters. The transfer learning dataset is split into training (70%), validation (20%) and test (10%) sets and trained for a maximum of 50 epochs. After each training epoch, we run a test epoch for assessing the test loss and data-specific test metrics. AlphaDIA uses a custom learning rate scheduler with two phases. The first phase is a warm-up period (default: five epochs) during which the learning rate gradually increases to a maximum value (default: 0.005). After this warm-up phase, the learning rate scheduler halves the learning rate if the training loss does not notably improve (default: >5% test loss) within a patience period (default: three epochs). Additionally, we use a simple early stopping mechanism that interrupts training if the validation loss starts to diverge or does not notably improve (default: 12 epochs).

After training, the deep learning model is stored on disk and can be loaded as necessary. Retention time and ion mobility fine-tuning are supervised by calculating the *L*_1_ loss, *R*^2^ and 95th percentile of the absolute error on the training data. MS2 fine-tuning is supervised by calculating the *L*_1_ loss, Pearson correlation coefficient, spectral angle and Spearman correlation on the test data. Charge fine-tuning is supervised by calculating the cross-entropy loss, accuracy, precision and recall on the test data. All training and test metrics are reported to the user. The specific implementation and details of the test metrics can be found in the open-source code on GitHub (www.github.com/MannLabs/alphadia).

### Sample preparation of HeLa bulk digests

HeLa S3 cells (American Type Culture Collection) were cultured in DMEM (Life Technologies) supplemented with 20 mM glutamine, 10% FBS and 1% penicillin–streptomycin. After washing the cells in PBS and cell lysis, the proteins were reduced, alkylated and digested by trypsin (Sigma-Aldrich) and LysC (WAKO) (1:100 enzyme to protein, w/w) in one step. The peptides were dried and resuspended in 0.1% trifluoroacetic acid and 2% acetonitrile; then, 200 ng of digest was loaded onto Evotips (Evosep). The Evotips were prepared by activation with 1-propanol, washed with 0.1% formic acid (FA) and 99.9% acetonitrile and equilibrated with 0.1% FA. After loading the samples, tips were washed once with 0.1% FA.

### Sample preparation of dimethylated peptides for transfer learning

HeLa cells were cultured as described above. A HeLa cell pellet was lysed by boiling for 10 min in 1% SDC in 60 mM TEAB pH 8.5, followed by sonication in a Branson type instrument, Heinemann Sonifier 250 (Schwäbisch Gmünd), operating at 20% duty cycle and 3–4 outputs for 1 min and boiling for 5 min again. After cooling to room temperature, the protein concentration was determined using the tryptophan fluorescence-based WF assay in the microtiter plate format using white Nunc 96-well plates with a flat bottom (Thermo Fisher Scientific, 136101). After diluting the lysate to 1 μg μl^−1^ in lysis buffer, disulfide bonds were reduced by adding TCEP to a final concentration of 10 mM and briefly incubating for 10 min. Denatured protein lysate was digested by ArgC Ultra (Promega) and LysC (WAKO) at 1:250 and 1:100 (enzyme to protein) ratios to the lysate at 37 °C for 3 h, respectively. The peptides were labeled with a dimethyl group using 100 μl of 1 μg μl^−1^ digested peptides and adding 4 μl of 4% formaldehyde and 4 μl of 0.6 M NaBH_3_CN solution. The mixture was incubated at room temperature and, every 10 min, 2.8 μl (2 μg of peptides) was sampled until 60 min and added to 17.2 μl of a 1 % solution of trifluoroacetic acid to quench the reaction.

### Sample preparation for the mixed-species experiments

For the mixed-species experiment, three different mixtures with varying mixing ratios of HeLa tryptic digest (Pierce, 1862824), *Saccharomyces cerevisiae* tryptic digest (Promega, V746A) and *Escherichia coli* tryptic digest (Waters, 186003196) were prepared: sample A, 10:1:10 human, yeast and *E.* *coli*; sample B, 10:10:1 human, yeast and *E.* *coli*; sample C, 10:4:7 human, yeast and *E.* *coli*. Five replicates containing 210 ng were loaded per condition.

### Peptide loading onto C-18 tips

C-18 tips (Evotip Pure, Evosep) were loaded with the Bravo robot (Agilent), followed by activation with 1‐propanol, washing two times with 50 μl buffer B (99.9% acetonitrile and 0.1% FA), activation with 1‐propanol and two wash steps with 50 μl of buffer A (99.9% H_2_O and 0.1% FA). In between, Evotips were spun at 700*g* for 1 min. For sample loading, Evotips were prepared with 70 μl of buffer A and a short spin at 700*g*. Samples were loaded in 20 μl with the indicated concentration into the remaining buffer A and spun at 700*g* for 1 min, unless described otherwise. After sample loading, Evotips were washed with 50 μl of buffer A and stored with 150 μl of buffer A after a short spin at 700*g* at 4 °C until MS acquisition.

### MS data acquisition of dia-PASEF and synchro-PASEF data

We used the Evosep One LC system to separate peptide mixtures at varying throughputs using standardized gradients. These gradients consisted of 0.1% FA and 99.9% water (v/v) and 0.1% FA with 99.9% acetonitrile (v/v) as the mobile phases. For the 60-SPD runs, peptides were separated on a Pepsep column (8 cm × 150 μm inner diameter, 1.5 μm C18; Bruker Daltonics) connected to a 10-μm (inner diameter) fused silica emitter (Bruker Daltonics). For the Whisper 40-SPD runs, we used an Aurora Elite nanoflow column (15 cm × 75 μm inner diameter, 1.7 μm C18; IonOpticks).

The system was coupled with a timsTOF MS instrument (Bruker Daltonics) to acquire data in dia-PASEF and synchro-PASEF modes. Sample loads above 25 ng were analyzed using a timsTOF Pro2 and those below 25 ng were analyzed using a timsTOF Ultra. The dia-PASEF and synchro-PASEF methods were optimized using our Python tool, py_diAID^39^. This tool maximizes precursor coverage by optimally positioning the acquisition scheme over the precursor cloud and enhances sampling efficiency by adjusting the isolation window widths according to precursor density.

The dia-PASEF method covers an *m*/*z* range from 300 to 1,200 with eight dia-PASEF scans and two isolation window positions per scan (cycle time: 0.98 s). The synchro-PASEF method covers an *m*/*z* range from 140 to 1,350 with four diagonal synchro scans (cycle time: 0.53 s). The method files are deposited to the data repository. In both modes, the fragment scans are acquired with an *m*/*z* range from 100 to 1,700. Furthermore, ions are accumulated and ejected at 100-ms intervals from the TIMS tunnel. The methods cover an ion mobility range from 1.3 to 0.7 V cm^−2^, calibrated with Agilent ESI tuning mix ions (*m*/*z*, 1/*K*_0_: 622.02, 0.98 V cm^−2^; 922.01, 1.19 V cm^−2^; 1221.99, 1.38 V cm^−2^). The collision energy was linearly decreased in relation to the ion mobility elution, from 59 eV at an ion mobility of 1.6 V cm^−2^ to 20 eV at 0.6 V cm^−2^.

### MS data acquisition of SWATH data on the Sciex 7600

Triplicates of 200-ng HeLa bulk digest were loaded onto C-18 tips as described above and analyzed using an Evosep One system (Evosep) coupled to a 7600 ZenoTOF MS instrument (Sciex) using Sciex OS (version 3.3 or higher). Peptides were separated by the 60-SPD method gradient (Evosep) on a PepSep reverse-phase column (8 cm × 150 μm) packed with 1.5 μm of C18 beads (Bruker Daltonics) at 50 °C connected to the low micro electrode for 1–10 μl min^−1^. The mobile phases were 0.1% FA in LC–MS-grade water (buffer A) and 99.9% acetonitrile and 0.1% FA (buffer B). The ZenoTOF MS instrument was equipped with the Optiflow ion source using a spray voltage of 4.5 kV, ion source gas 1 of 15 psi, ion source gas 2 of 60 psi, curtain gas of 35 psi, collision-activated dissociation gas of 7 and a temperature of 200 °C. SWATH data were acquired using the following parameters: TOF MS start mass of 400 Da, stop mass of 1,500 Da, TOF MS accumulation time of 50 ms, TOF MSMS start mass of 140 Da, stop mass of 1750 Da, accumulation time of 13 ms with dynamic collision energy turned on, a charge state of 2, Zeno pulsing enabled and 60 variable SWATH windows covering the mass range of 400–900 *m*/*z*.

### MS data acquisition of mixed-species samples on the Orbitrap Astral

For mixed-species experiments, five replicates of samples A, B and C were loaded onto C-18 tips as described above. Samples were analyzed using an Evosep One system (Evosep) coupled to a Orbitrap Astral MS instrument (Thermo Scientific) using Thermo Tune software (version 1.0 or higher). Peptides were separated by the 60-SPD method gradient (Evosep) on a PepSep reverse-phase column (8 cm × 150 μm) packed with 1.5 μm of C18 beads (Bruker Daltonics) at 50 °C. The analytical column was connected to a stainless-steel emitter with inner diameter of 30 µm (EV1086). The mobile phases were 0.1% FA in LC–MS-grade water (buffer A) and 99.9% acetonitrile and 0.1% FA (buffer B). The Orbitrap Astral MS instrument was equipped with a FAIMS Pro interface and an EASY-Spray source (both Thermo Scientific). A compensation voltage of −40 V and a total carrier gas flow of 3.5 L min^−1^ were used and an electrospray voltage of 2.0 kV was applied for ionization. The MS1 spectra were recorded using the Orbitrap analyzer at 120,000 resolution from *m*/*z* 380 to 980 using an automatic gain control (AGC) target of 500% and a maximum injection time of 3 ms. The Astral analyzer was used for MS/MS scans in data-independent mode with 3-Th nonoverlapping isolation windows with a scan range of 150–2,000 *m*/*z*. The precursor accumulation time was 3 ms with an AGC target of 500%. The isolated ions were fragmented using higher-energy collision dissociation (HCD) with 25% normalized collision energy (NCE).

### MS data acquisition of HeLa bulk data on the Orbitrap Astral

For analysis of HeLa bulk digest, 200 ng of lysate was loaded onto C-18 tips in six replicates as described above. Samples were analyzed using an Evosep One system (Evosep) coupled to a Orbitrap Astral MS instrument (Thermo Scientific) using Thermo Tune software (version 1.0 or higher). Peptides were separated by the 60-SPD method gradient (Evosep) on an Aurora Rapid reverse-phase column (80 mm × 0.15 mm) packed with 1.7 μm of C18 beads (IonOpticks) at 50 °C. The mobile phases were 0.1% FA in LC–MS-grade water (buffer A) and 99.9% acetonitrile and 0.1% FA (buffer B). The Orbitrap Astral MS instrument was equipped with a FAIMS Pro interface and an EASY-Spray source (both Thermo Scientific). A compensation voltage of −40 V and a total carrier gas flow of 3.5 L min^−1^ were used and an electrospray voltage of 1.9 kV was applied for ionization. The MS1 spectra were recorded using the Orbitrap analyzer at 120,000 resolution from *m*/*z* 380 to 980 using an AGC target of 500% and a maximum injection time of 3 ms. The Astral analyzer was used for MS/MS scans in data-independent mode with 2-Th nonoverlapping isolation windows with a scan range of 150–2000 *m*/*z*. The precursor accumulation time was 3 ms with an AGC target of 500%. The isolated ions were fragmented using HCD with 25% NCE.

### MS data acquisition of dimethylated peptides on the Orbitrap Astral

MS data acquisition was performed as described for mixed-species samples on the Orbitrap Astral, unless described otherwise. For each of the six timepoints, triplicates of 50 ng of labeled peptide were injected. Samples were separated by the Whisper 40-SPD method gradient (Evosep) on an Aurora Elite TS column (15 cm, 75 µm inner diameter; AUR3-15075C18-TS, IonOpticks) at 50 °C. An electrospray voltage of 1.9 kV was applied. The MS1 resolution was 240,000 with a maximum injection time of 100 ms and 6 ms for MS/MS.

### Search and analysis of dia-PASEF and synchro-PASEF data with alphaDIA

Data were searched with version 1.5.5 of alphaDIA using a previously published^39^ empirical HeLa library. A default single-step search was used with the following parameters: target MS1 tolerance, 15 ppm; target MS2 tolerance, 15 ppm; number of target candidates, 5. For synchro-PASEF, quant_all = true was set and a quant_window of six scans was used. All precursors with run-level FDR of 1% and protein groups with a global FDR of 1% were accepted. CVs were calculated on non-log-transformed directLFQ-normalized quantities.

### Search and analysis of ZenoTOF data with alphaDIA

Data were searched with version 1.5.5 of alphaDIA using the HeLa library mentioned above. A default single-step search was used with the following parameters: target MS1 tolerance, 15 ppm; target MS2 tolerance, 15 ppm; number of target candidates, 3; target retention time tolerance, 300 s. All precursors with run-level FDR of 1% and protein groups with global FDR of 1% were accepted. CVs were calculated on non-log-transformed directLFQ-normalized quantities.

### Search and analysis of empirical library data from Lou et al.

Raw files, libraries and FASTA files were used as provided in the original publication^41^. All data were searched with alphaDIA 1.5.5 using default parameters. For timsTOF data, the following parameters were changed: target MS1 tolerance, 15 ppm; target MS2 tolerance, 15 ppm; number of target candidates; quant_window, 6; group level, genes, scans; target retention time tolerance, 500 s. For QE-HF, the data search was performed with a target MS1 tolerance of 5 ppm, target MS2 tolerance of 10 ppm, five target candidates, a quant_window of six scans, group level of genes and scans and a target retention time tolerance of 600 s. Data for benchmarked tools were used as provided in the original publication. Analysis was performed as described in the original publication except for reassignment of proteins. Instead, search-engine-specific protein grouping was used. For alphaDIA, precursors passing a local 1% FDR and protein groups passing a global 1% FDR were accepted.

### Search and analysis of HeLa bulk data with fully predicted spectral libraries

For fully predicted library benchmarking, Spectronaut version 18.6.231227.55695, DIA-NN version 2.1.0, CHIMERYS^53^ version 4.2.1 and alphaDIA version 1.10.2 were used. All analysis was performed using the same FASTA file of reviewed human proteins without isoforms (December 1, 2023). On all platforms, the search was performed for tryptic precursors with carbamidomethyl modification at cysteine as a fixed modification and variable methionine oxidation and protein N-terminal acetylation with a maximum of two occurrences. Charge states of 2–4 were included with sequence lengths between 7 and 35 aa with a single missed cleavage. For CHIMERYS, only peptides with up to 30 aa were used as the tool does not support 35 aa. For alphaDIA, automatic library prediction by alphaPeptDeep was used with the Lumos model for an NCE of 25. AlphaDIA used default parameters for a two-step search with the following changes: target MS1 tolerance, 4 ppm; target MS2 tolerance, 7 ppm. All data were analyzed at a 1% FDR threshold as enforced by the search engine. CVs were calculated on non-log-transformed intensities as provided by the search engine for all proteins.

For entrapment analysis, an *Arabidopsis* FASTA with reviewed sequences and no isoforms was downloaded from UniProt (February 2, 2024). The search was performed as described above with heuristic inference. After the search, all shared precursors including isoleucine–leucine pairs were identified. Protein groups with shared precursors were discarded.

### Search and analysis of mixed-species data with fully predicted spectral libraries

For all three species, reviewed nonisoform proteomes were downloaded from UniProt (February 21, 2024). Proteins were in silico digested using tryptic cleavage with carbamidomethyl modification at cysteine as a fixed modification and variable methionine oxidation and protein N-terminal acetylation with a maximum of two occurrences. Charge states of 2–4 were included with sequence lengths between 7 and 35 aa with a single missed cleavage. The library was predicted using the alphaPeptDeep Lumos model at 25 NCE. AlphaDIA 1.5.4 was used with default parameters for a two-step search with the following changes: number of target candidates, 5; target MS1 tolerance, 5 ppm; target MS2 tolerance, 10 ppm; target retention time tolerance, 200 s for the first pass and 100 s for the second pass. Heuristic protein inference was used on the gene level. Proteins with shared sequences were removed as described above. For benchmarking accuracy, the median LFQ ratio was calculated for protein groups identified in at least three replicates.

### Search and analysis of SILAC data with fully predicted spectral libraries

Data were searched with version 1.5.5 of alphaDIA. A fully predicted human library was generated with alphaPeptDeep as described above but for an NCE of 27. The library was multiplexed across the light channel without additional modifications and a heavy channel with isotopic labeling of arginine (+10.008269) and lysine (+8.014199). A single-step search was performed using alphaDIA with default parameters other than the following changes: target MS1 tolerance, 5 ppm; target MS2 tolerance, 20 ppm; target retention time tolerance, 600 s; channel_wise_fdr = true.

### Search and analysis of dimethylated samples using transfer learning

A fully predicted human library was generated on the basis of a reviewed human UniProt library (December 1, 2023) with the general pretrained alphaPeptDeep model not trained on dimethylated peptides. The peptides were modified with methionine oxidation and protein N-terminal acetylation as variable modifications with a maximum of two. N-terminal and lysine dimethylation were set as fixed modifications. Transfer search was performed using alphaDIA 1.5.5 with default parameters other than the following changes: number of target candidates, 1; target MS1 tolerance, 4 ppm; target MS2 tolerance, 7 ppm; target retention time tolerance, 1,200 s. Transfer learning quantification was enabled and set to *b* and *y* ions with a maximum charge of 2 and the top three occurrences for every modified sequence. The generated transfer learning library was used for training with the default training scheme described above. For evaluation, the original pretrained model, the transfer learned retention time model, the transfer learned MS2 model and the fully transfer learned model were evaluated for search. All searches were performed with the same parameters as the transfer search apart from a target retention time tolerance of 100 s for searches with the updated model.

### Search and analysis of transfer learning entrapments

For evaluation of transfer learning on FDRs, entrapment experiments with known false-positive *Arabidopsis* peptides were performed on the unmodified HeLa bulk samples acquired on the Orbitrap Astral. The entrapment library was generated as described above for the two-step search with N-terminal glutamate and glutamine to pyroglutamate conversion added as variable modifications. Raw files were searched with alphaDIA 1.5.5 using default parameters other than the following changes: number of target candidates, 1; target MS1 tolerance, 4 ppm; target MS2 tolerance, 7 ppm; target retention time tolerance, 1,200 s. Transfer learning quantification was enabled and set to *b* and *y* ions with a maximum charge of 2 and the top three occurrences for every modified sequence. Transfer learning was performed using all human and *Arabidopsis* precursors identified at the 1% FDR cutoff. The transfer learning model was then reused for a second search with an updated target retention time tolerance of 150 s. The process was repeated twice and the identifications after every search were analyzed for the number of false-positive *Arabidopsis* identifications as described above.

### Data analysis and plotting

All analyses were performed using Python 3.11.11 on macOS 14.3.0. Data manipulation and analysis were conducted using pandas 2.2.3, NumPy 1.26.4 and SciPy 1.15.2. Statistical analysis and machine learning were performed using scikit-learn 1.6.1. Data visualization was created using matplotlib 3.9.0 and seaborn 0.13.2. Unless specified otherwise, box plots extend from the first quartile (Q1) to the third quartile (Q3) with the median shown as line. Whiskers extend from 1.5 times the interquartile range below Q1 to 1.5 times the interquartile range above Q3.

### Reporting summary

Further information on research design is available in the Nature Portfolio Reporting Summary linked to this article.

## Online content

Any methods, additional references, Nature Portfolio reporting summaries, source data, extended data, supplementary information, acknowledgements, peer review information; details of author contributions and competing interests; and statements of data and code availability are available at 10.1038/s41587-025-02791-w.

## Supplementary information

Supplementary Information Supplementary Figs. 1–5.

Reporting Summary

## Data Availability

All raw data and search results were deposited to the ProteomeXchange Consortium repository with the MassIVE identifiers MSV000095138 and MSV000098448. Original benchmarking data for library search as used from Lou et al.^41^ were obtained from ProteomeXchange with identifier PXD034709.

## Code availability

All code presented herein as part of alphaDIA is free software accessible under the permissive Apache license. Source code for AlphaDIA (www.github.com/MannLabs/alphadia), alphaRaw (www.github.com/MannLabs/alpharaw) and alphaBase (www.github.com/MannLabs/alphabase) can be found on GitHub.

## References

[1] Aebersold, R. & Mann, M. Mass-spectrometric exploration of proteome structure and function. *Nature* **537**, 347–355 (2016). 27629641 10.1038/nature19949

[2] Navarro, P. et al. A multicenter study benchmarks software tools for label-free proteome quantification. *Nat. Biotechnol.* **34**, 1130–1136 (2016). 27701404 10.1038/nbt.3685 PMC5120688

[3] Cox, J. & Mann, M. MaxQuant enables high peptide identification rates, individualized p.p.b.-range mass accuracies and proteome-wide protein quantification. *Nat. Biotechnol.* **26**, 1367–1372 (2008). 19029910 10.1038/nbt.1511

[4] Kong, A. T., Leprevost, F. V., Avtonomov, D. M., Mellacheruvu, D. & Nesvizhskii, A. I. MSFragger: ultrafast and comprehensive peptide identification in mass spectrometry-based proteomics. *Nat. Methods* **14**, 513–520 (2017). 28394336 10.1038/nmeth.4256 PMC5409104

[5] Lazear, M. R. Sage: an open-source tool for fast proteomics searching and quantification at scale. *J. Proteome Res.* **22**, 3652–3659 (2023). 37819886 10.1021/acs.jproteome.3c00486

[6] O’Reilly, F. J. & Rappsilber, J. Cross-linking mass spectrometry: methods and applications in structural, molecular and systems biology. *Nat. Struct. Mol. Biol.* **25**, 1000–1008 (2018). 30374081 10.1038/s41594-018-0147-0

[7] Virág, D. et al. Current trends in the analysis of post-translational modifications. *Chromatographia* **83**, 1–10 (2020).

[8] Liu, H., Sadygov, R. G. & Yates, J. R. A model for random sampling and estimation of relative protein abundance in shotgun proteomics. *Anal. Chem.* **76**, 4193–4201 (2004). 15253663 10.1021/ac0498563

[9] Gillet, L. C. et al. Targeted data extraction of the MS/MS spectra generated by data-independent acquisition: a new concept for consistent and accurate proteome analysis. *Mol. Cell. Proteomics* **11**, O111.016717 (2012). 22261725 10.1074/mcp.O111.016717 PMC3433915

[10] Collins, B. C. et al. Multi-laboratory assessment of reproducibility, qualitative and quantitative performance of SWATH-mass spectrometry. *Nat. Commun.* **8**, 291 (2017). 28827567 10.1038/s41467-017-00249-5 PMC5566333

[11] Messner, C. B. et al. Ultra-fast proteomics with Scanning SWATH. *Nat. Biotechnol.* **39**, 846–854 (2021). 33767396 10.1038/s41587-021-00860-4 PMC7611254

[12] Brunner, A. et al. Ultra‐high sensitivity mass spectrometry quantifies single‐cell proteome changes upon perturbation. *Mol. Syst. Biol.* **18**, e10798 (2022). 35226415 10.15252/msb.202110798 PMC8884154

[13] Bernhardt, O. et al. Spectronaut: a fast and efficient algorithm for MRM-like processing of data independent acquisition (SWATH-MS) data. In *Proceedings of the 60th ASMS Conference on Mass Spectrometry and Allied Topics* (ASMS, 2012).

[14] Tsou, C.-C. et al. DIA-Umpire: comprehensive computational framework for data-independent acquisition proteomics. *Nat. Methods* **12**, 258–264 (2015). 25599550 10.1038/nmeth.3255 PMC4399776

[15] Demichev, V., Messner, C. B., Vernardis, S. I., Lilley, K. S. & Ralser, M. DIA-NN: neural networks and interference correction enable deep proteome coverage in high throughput. *Nat. Methods* **17**, 41–44 (2020). 31768060 10.1038/s41592-019-0638-x PMC6949130

[16] Searle, B. C. et al. Chromatogram libraries improve peptide detection and quantification by data independent acquisition mass spectrometry. *Nat. Commun.* **9**, 5128 (2018). 30510204 10.1038/s41467-018-07454-w PMC6277451

[17] Sinitcyn, P. et al. MaxDIA enables library-based and library-free data-independent acquisition proteomics. *Nat. Biotechnol.* **39**, 1563–1573 (2021). 34239088 10.1038/s41587-021-00968-7 PMC8668435

[18] Cox, J. Prediction of peptide mass spectral libraries with machine learning. *Nat. Biotechnol.* **41**, 33–43 (2023). 36008611 10.1038/s41587-022-01424-w

[19] Gessulat, S. et al. Prosit: proteome-wide prediction of peptide tandem mass spectra by deep learning. *Nat. Methods* **16**, 509–518 (2019). 31133760 10.1038/s41592-019-0426-7

[20] Zeng, W.-F. et al. AlphaPeptDeep: a modular deep learning framework to predict peptide properties for proteomics. *Nat. Commun.* **13**, 7238 (2022). 36433986 10.1038/s41467-022-34904-3 PMC9700817

[21] Bouwmeester, R., Gabriels, R., Hulstaert, N., Martens, L. & Degroeve, S. DeepLC can predict retention times for peptides that carry as-yet unseen modifications. *Nat. Methods* **18**, 1363–1369 (2021). 34711972 10.1038/s41592-021-01301-5

[22] Bekker-Jensen, D. B. et al. Rapid and site-specific deep phosphoproteome profiling by data-independent acquisition without the need for spectral libraries. *Nat. Commun.* **11**, 787 (2020). 32034161 10.1038/s41467-020-14609-1 PMC7005859

[23] Steger, M. et al. Time-resolved in vivo ubiquitinome profiling by DIA-MS reveals USP7 targets on a proteome-wide scale. *Nat. Commun.* **12**, 5399 (2021). 34518535 10.1038/s41467-021-25454-1 PMC8438043

[24] Dens, C., Yeung, D., Krokhin, O., Laukens, K. & Bittremieux, W. Zero-shot retention time prediction for unseen post-translational modifications with molecular structure encodings. Preprint at *bioRxiv*10.1101/2024.12.18.629045 (2024).

[25] Gao, M. et al. Deep representation features from DreamDIAXMBD improve the analysis of data-independent acquisition proteomics. *Commun. Biol.* **4**, 1190 (2021). 34650228 10.1038/s42003-021-02726-6 PMC8517002

[26] Song, J. & Yu, C. Alpha-Tri: a deep neural network for scoring the similarity between predicted and measured spectra improves peptide identification of DIA data. *Bioinformatics* **38**, 1525–1531 (2022). 34999750 10.1093/bioinformatics/btab878

[27] Peckner, R. et al. Specter: linear deconvolution for targeted analysis of data-independent acquisition mass spectrometry proteomics. *Nat. Methods* **15**, 371–378 (2018). 29608554 10.1038/nmeth.4643 PMC5924490

[28] Guzman, U. H. et al. Ultra-fast label-free quantification and comprehensive proteome coverage with narrow-window data-independent acquisition. *Nat. Biotechnol.* **42**, 1855–1866 (2024). 38302753 10.1038/s41587-023-02099-7 PMC11631760

[29] Wang, Z. et al. High-throughput proteomics of nanogram-scale samples with Zeno SWATH MS. *eLife* **11**, e83947 (2022). 36449390 10.7554/eLife.83947 PMC9711518

[30] Meier, F. et al. diaPASEF: parallel accumulation–serial fragmentation combined with data-independent acquisition. *Nat. Methods* **17**, 1229–1236 (2020). 33257825 10.1038/s41592-020-00998-0

[31] Demichev, V. et al. dia-PASEF data analysis using FragPipe and DIA-NN for deep proteomics of low sample amounts. *Nat. Commun.* **13**, 3944 (2022). 35803928 10.1038/s41467-022-31492-0 PMC9270362

[32] Distler, U. et al. midiaPASEF maximizes information content in data-independent acquisition proteomics. Preprint at *bioRxiv*10.1101/2023.01.30.526204 (2023).

[33] Skowronek, P. et al. Synchro-PASEF allows precursor-specific fragment ion extraction and interference removal in data-independent acquisition. *Mol. Cell. Proteomics* **22**, 100489 (2023). 36566012 10.1016/j.mcpro.2022.100489 PMC9868879

[34] Below, C. R. et al. Enhanced identifications and quantification through retention time down-sampling in fast-cycling diagonal-PASEF methods. Preprint at *bioRxiv*10.1101/2025.04.23.650190 (2025). 10.1016/j.mcpro.2025.101480 PMC12818240 41380996

[35] Skowronek, P., Wallmann, G., Wahle, M., Willems, S. & Mann, M. An accessible workflow for high-sensitivity proteomics using parallel accumulation–serial fragmentation. *Nat. Protoc.* **20**, 1700–1729 (2025). 39825144 10.1038/s41596-024-01104-w

[36] Zeng, W.-F. et al. MS/MS spectrum prediction for modified peptides using pDeep2 trained by transfer learning. *Anal. Chem.* **91**, 9724–9731 (2019). 31283184 10.1021/acs.analchem.9b01262

[37] Liu, Z. et al. DIA-BERT: pre-trained end-to-end transformer models for enhanced DIA proteomics data analysis. *Nat. Commun.* **16**, 3530 (2025). 40229248 10.1038/s41467-025-58866-4 PMC11997033

[38] Strauss, M. T. et al. AlphaPept: a modern and open framework for MS-based proteomics. *Nat. Commun.* **15**, 2168 (2024). 38461149 10.1038/s41467-024-46485-4 PMC10924963

[39] Skowronek, P. et al. Rapid and in-depth coverage of the (phospho-)proteome with deep libraries and optimal window design for dia-PASEF. *Mol. Cell. Proteomics* **21**, 100279 (2022). 35944843 10.1016/j.mcpro.2022.100279 PMC9465115

[40] Ammar, C., Schessner, J. P., Willems, S., Michaelis, A. C. & Mann, M. Accurate label-free quantification by directLFQ to compare unlimited numbers of proteomes. *Mol. Cell. Proteomics* **22**, 100581 (2023). 37225017 10.1016/j.mcpro.2023.100581 PMC10315922

[41] Lou, R. et al. Benchmarking commonly used software suites and analysis workflows for DIA proteomics and phosphoproteomics. *Nat. Commun.* **14**, 94 (2023). 36609502 10.1038/s41467-022-35740-1 PMC9822986

[42] Huang, T., Wang, J., Yu, W. & He, Z. Protein inference: a review. *Brief. Bioinform.* **13**, 586–614 (2012). 22373723 10.1093/bib/bbs004

[43] Granholm, V., Noble, W. S. & Käll, L. On using samples of known protein content to assess the statistical calibration of scores assigned to peptide-spectrum matches in shotgun proteomics. *J. Proteome Res.* **10**, 2671–2678 (2011). 21391616 10.1021/pr1012619 PMC3268674

[44] Cox, J. et al. Accurate proteome-wide label-free quantification by delayed normalization and maximal peptide ratio extraction, termed MaxLFQ. *Mol. Cell. Proteomics* **13**, 2513–2526 (2014). 24942700 10.1074/mcp.M113.031591 PMC4159666

[45] Derks, J. et al. Increasing the throughput of sensitive proteomics by plexDIA. *Nat. Biotechnol.* **41**, 50–59 (2023). 35835881 10.1038/s41587-022-01389-w PMC9839897

[46] Thielert, M. et al. Robust dimethyl‐based multiplex‐DIA doubles single‐cell proteome depth via a reference channel. *Mol. Syst. Biol.* **19**, e11503 (2023). 37602975 10.15252/msb.202211503 PMC10495816

[47] Pino, L. K., Baeza, J., Lauman, R., Schilling, B. & Garcia, B. A. Improved SILAC quantification with data-independent acquisition to investigate bortezomib-induced protein degradation. *J. Proteome Res.* **20**, 1918–1927 (2021). 33764077 10.1021/acs.jproteome.0c00938 PMC8256668

[48] Wen, B. et al. Carafe enables high quality in silico spectral library generation for data-independent acquisition proteomics. Preprint at *bioRxiv*10.1101/2024.10.15.618504 (2024). 10.1038/s41467-025-64928-4 PMC12592563 41198693

[49] Perez-Riverol, Y. et al. Open-source and FAIR research software for proteomics. *J. Proteome Res.* **24**, 2222–2234 (2025). 40267229 10.1021/acs.jproteome.4c01079 PMC12053954

[50] Willems, S., Voytik, E., Skowronek, P., Strauss, M. T. & Mann, M. AlphaTims: indexing trapped ion mobility spectrometry–TOF data for fast and easy accession and visualization. *Mol. Cell. Proteomics* **20**, 100149 (2021). 34543758 10.1016/j.mcpro.2021.100149 PMC8526765

[51] Nesvizhskii, A. I. A survey of computational methods and error rate estimation procedures for peptide and protein identification in shotgun proteomics. *J. Proteomics* **73**, 2092–2123 (2010). 20816881 10.1016/j.jprot.2010.08.009 PMC2956504

[52] Nesvizhskii, A. I. & Aebersold, R. Interpretation of shotgun proteomic data. *Mol. Cell. Proteomics* **4**, 1419–1440 (2005). 16009968 10.1074/mcp.R500012-MCP200

[53] Frejno, M. et al. Unifying the analysis of bottom-up proteomics data with CHIMERYS. *Nat. Methods* **22**, 1017–1027 (2025). 40263583 10.1038/s41592-025-02663-w PMC12074992

