# Supplementary material for: AlphaDIA enables DIA transfer learning for feature-free proteomics
Source: Nat Biotechnol. 2025 Oct 21;44(7):1168–77. doi: 10.1038/s41587-025-02791-w (PMC13368584; doi:10.1038/s41587-025-02791-w)
Supplement: Supplementary file 1 — Supplementary Figs. 1–5. [file 41587_2025_2791_MOESM1_ESM.pdf]

nature biotechnology

Supplementary information https://doi.org/10.1038/s41587-025-02791-w

AlphaDIA enables DIA transfer learning for feature-free proteomics

In the format provided by the authors and unedited

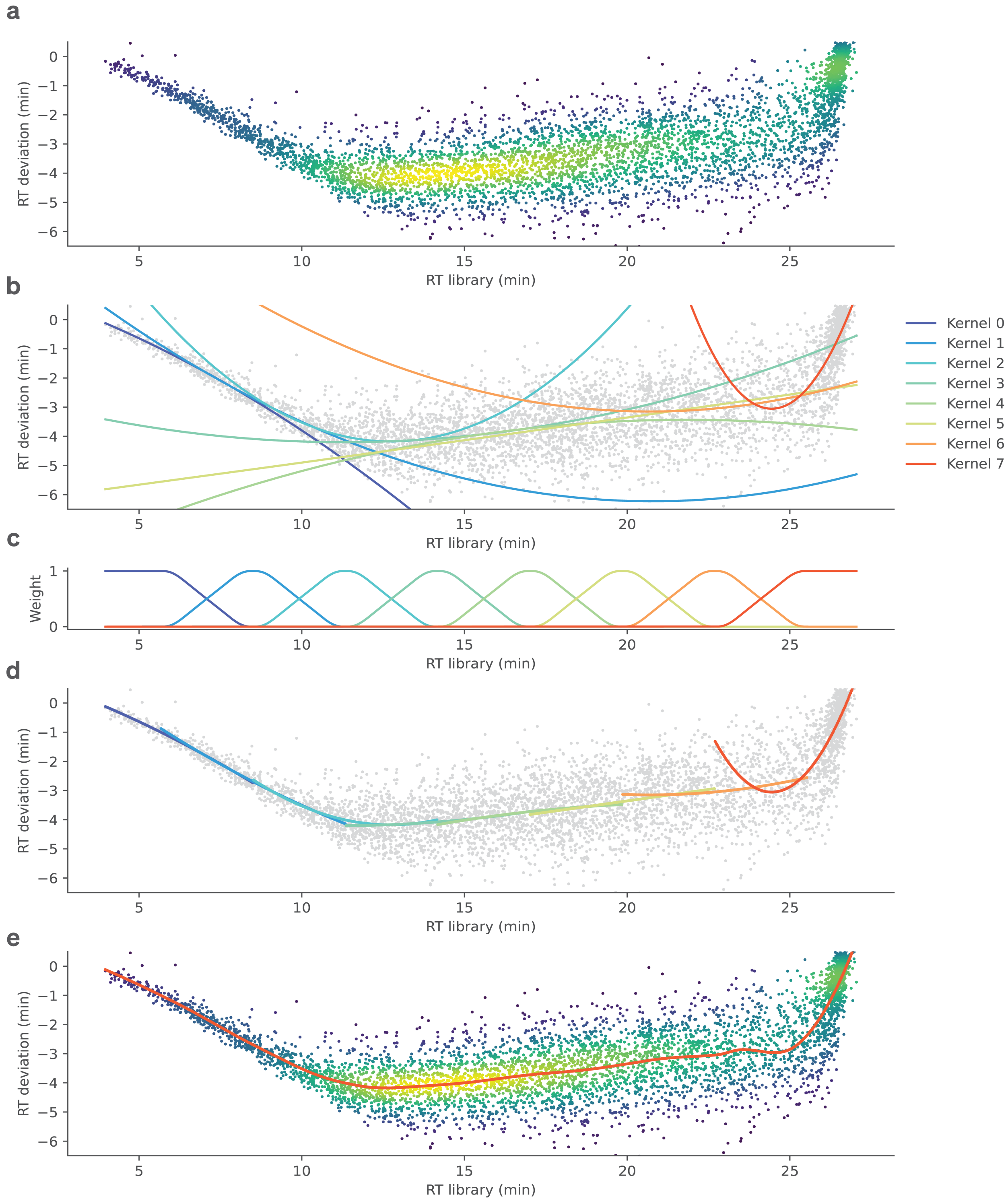

Supplementary Fig. 1 | Calibration of library properties to observed data using locally estimated scatterplot smoothing (LOESS) regression. a, Observed retention times of confidently identified precursors compared with the library annotated values. The absolute deviation in minutes is shown. **b**, A collection of polynomial kernels is fitted to uniformly distributed subregions of the data. **c**, The functions are combined and smoothed using tricubic weights. **d**, Combining the

kernels with their weighting functions allows to approximate the systematic deviation of the data locally. **e**, The sum of the weighted kernels can then be used for continuous approximation and calibration of retention times.

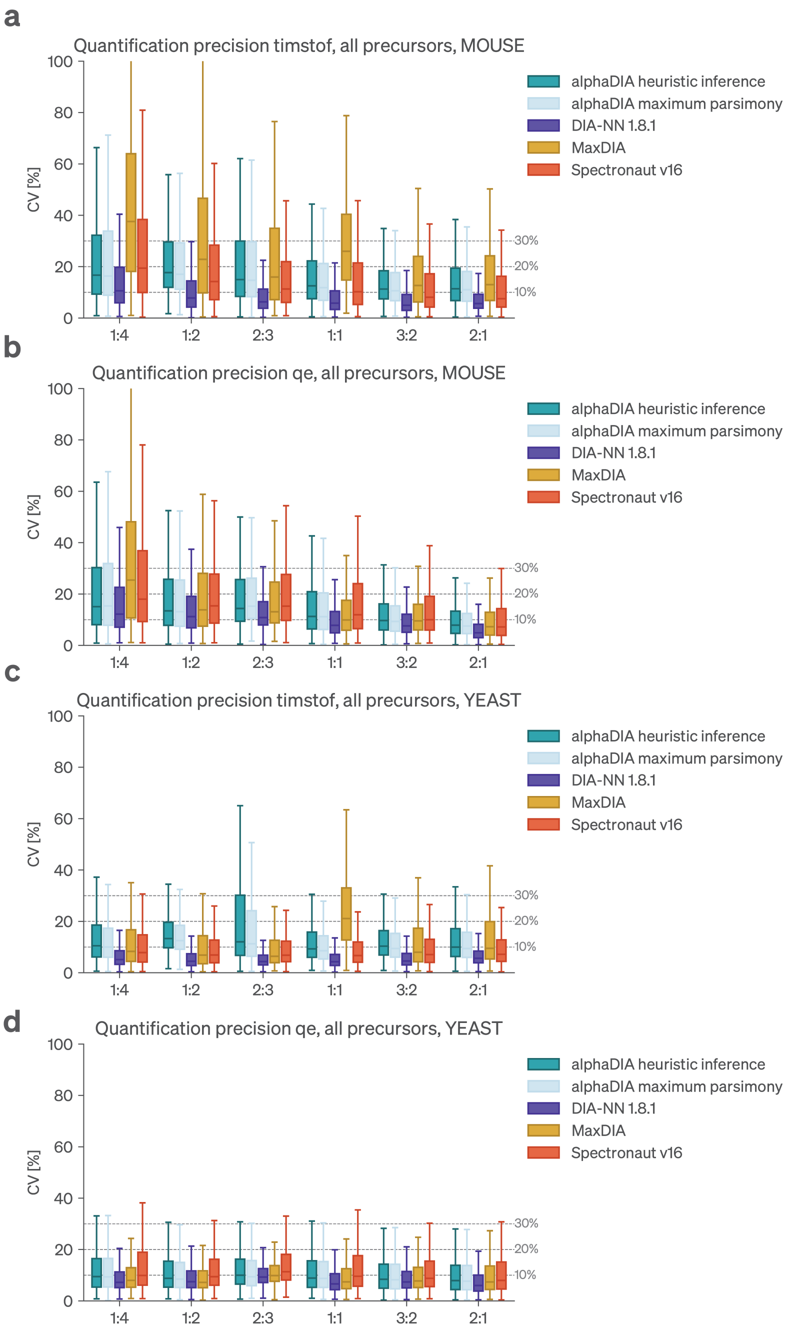

Supplementary Fig. 2 | Coefficient of variation for proteins in the empirical library benchmark. The quantitative precision was assessed by calculating the coefficient of variation for quantifiable protein abundances, identified in at least three out of five replicates (boxplot defined as per Error! Reference source not found.). **a**, Mouse proteins identified on the timsTOF. **b** Mouse proteins identified on the QE-HF **c**, Yeast proteins identified on the timsTOF. **d**, Yeast proteins identified on the QE-HF.

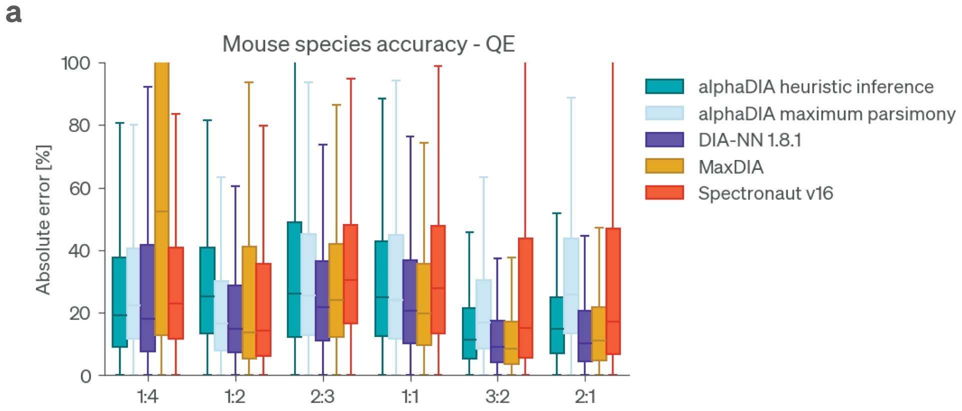

Supplementary Fig. 3 | Quantitative accuracy for ratios in the benchmarking dataset. a Ratios were calculated as described in the original study for proteins quantified in at least three out of five replicates. The absolute error between the expected and observed ratio is shown for different search engines (boxplot defined as per Error! Reference source not found.).

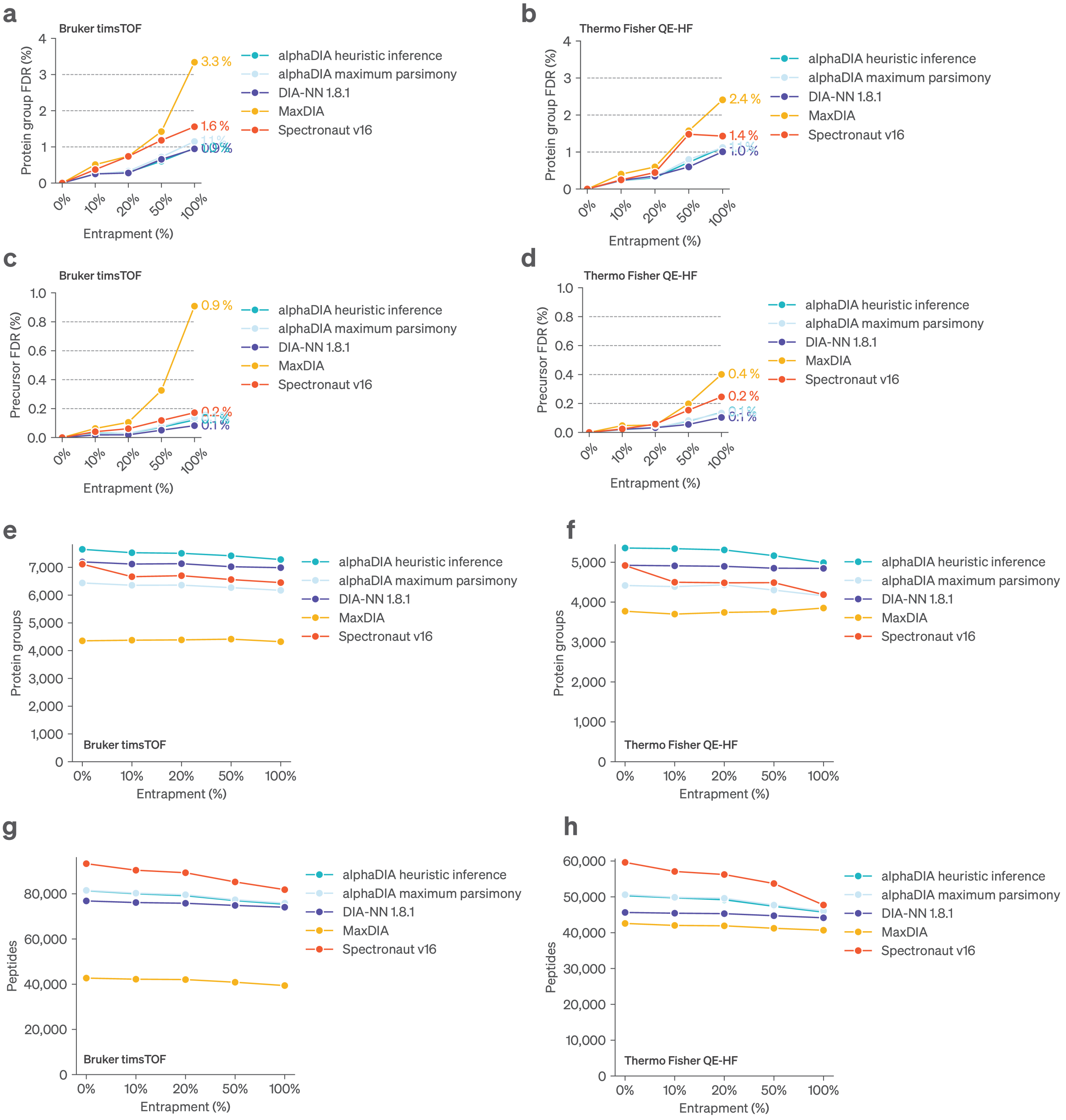

Supplementary Fig. 4 | FDR benchmarking using Arabidopsis entrapments. Target Mouse and Yeast libraries were spiked in with increasing amounts of known false positive Arabidopsis precursors as provided by Lou et al.[33] **a-d**, Number of global known false positive Arabidopsis proteins as a fraction of all identified proteins is shown as entrapment FDR. Search results are shown for increasing amounts of entrapment precursors, relative to the target library. **a**, Benchmarking data acquired on timsTOF, entrapment FDR calculated on the protein group level. **b**, Benchmarking data acquired on QE-HF, entrapment FDR calculated on the protein group level. **c**, Benchmarking data acquired on timsTOF, entrapment FDR calculated on the precursor level. **d**, Benchmarking data acquired on QE-HF, entrapment FDR calculated on the precursor level. **e-f**, Corresponding absolute protein and peptide numbers identified at 1% FDR when searched with increasing entrapment libraries. **e**, Identified protein groups on the timsTOF. **f**, Identified Proteins on the QE-HF. **g**, Identified Peptides on the timsTOF. **h**, Identified Peptides on the QE-HF.

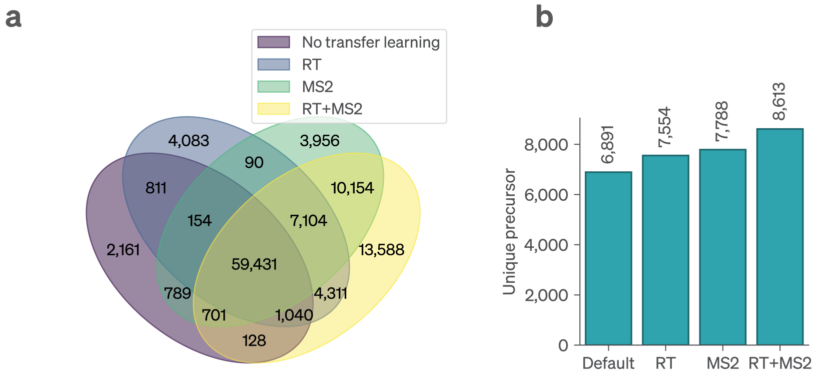

Supplementary Fig. 5 | Comparison of identification with transfer learning of dimethylation. a, Venn diagram showing the overlap of precursor identifications before and after transfer learning. **b**, Total number of unique protein groups identified across replicates after different stages of transfer learning.
