# Supplementary material for: AlphaDIA enables DIA transfer learning for feature-free proteomics
Source: Nat Biotechnol. 2025 Oct 21;44(7):1168–77. doi: 10.1038/s41587-025-02791-w (PMC13368584; doi:10.1038/s41587-025-02791-w)
Supplement: Supplementary file 2 — Reporting Summary [file 41587_2025_2791_MOESM2_ESM.pdf]

nature portfolio

Corresponding author(s): Matthias Mann, Wen-Feng Zeng

Last updated by author(s): Jul 8, 2025

Reporting Summary

Nature Portfolio wishes to improve the reproducibility of the work that we publish. This form provides structure for consistency and transparency in reporting. For further information on Nature Portfolio policies, see our Editorial Policies and the Editorial Policy Checklist.

Statistics

For all statistical analyses, confirm that the following items are present in the figure legend, table legend, main text, or Methods section.

n/a	Confirmed	
☐	☒	The exact sample size (*n*) for each experimental group/condition, given as a discrete number and unit of measurement
☐	☒	A statement on whether measurements were taken from distinct samples or whether the same sample was measured repeatedly
☒	☐	The statistical test(s) used AND whether they are one- or two-sided<br>*Only common tests should be described solely by name; describe more complex techniques in the Methods section.*
☒	☐	A description of all covariates tested
☒	☐	A description of any assumptions or corrections, such as tests of normality and adjustment for multiple comparisons
☐	☒	A full description of the statistical parameters including central tendency (e.g. means) or other basic estimates (e.g. regression coefficient) AND variation (e.g. standard deviation) or associated estimates of uncertainty (e.g. confidence intervals)
☒	☐	For null hypothesis testing, the test statistic (e.g. *F*, *t*, *r*) with confidence intervals, effect sizes, degrees of freedom and *P* value noted<br>*Give P values as exact values whenever suitable.*
☒	☐	For Bayesian analysis, information on the choice of priors and Markov chain Monte Carlo settings
☒	☐	For hierarchical and complex designs, identification of the appropriate level for tests and full reporting of outcomes
☐	☒	Estimates of effect sizes (e.g. Cohen's *d*, Pearson's *r*), indicating how they were calculated

Our web collection on statistics for biologists contains articles on many of the points above.

Software and code

Policy information about availability of computer code

Data collection	Thermo Tune version 1.0 or higher was used for data acquisition on Thermo Fisher instruments, Sciex OS 3.3 or higher was used for data acquisition on Sciex instruments. On Bruker instruments Bruker HyStar (v6.0), timsControl (v4.0.5_9ef8626_1) were used.
Data analysis	The software presented as part of this manuscript, alphaDIA is available at github.com/MannLabs/alphadia/. The following versions of alphaDIA were used with their use described in the methods section: alphadia v1.5.4,v1.5.5, v1.10.2. Further Spectronaut v18.6, DIA-NN 2.1.0 and Chimerys53 4.2.1 were used. All analyses were performed using Python 3.11.11 on macOS 14.3.0. Data manipulation and analysis were conducted using pandas 2.2.3, NumPy 1.26.4, and SciPy 1.15.2. Statistical analysis and machine learning were performed using scikit-learn 1.6.1. Data visualization was created using matplotlib 3.9.0 and seaborn 0.13.2.

For manuscripts utilizing custom algorithms or software that are central to the research but not yet described in published literature, software must be made available to editors and reviewers. We strongly encourage code deposition in a community repository (e.g. GitHub). See the Nature Portfolio guidelines for submitting code & software for further information.

Data

Policy information about availability of data

All manuscripts must include a data availability statement. This statement should provide the following information, where applicable:

- Accession codes, unique identifiers, or web links for publicly available datasets
- A description of any restrictions on data availability
- For clinical datasets or third party data, please ensure that the statement adheres to our policy

All raw data and search results have been deposited to the ProteomeXchange Consortium repository with the MassIVE identifier MSV000095138. Original benchmarking data for library search as used from lou et al. 2023 was used from Proteomexange with identifier PXD034709.

Research involving human participants, their data, or biological material

Policy information about studies with human participants or human data. See also policy information about sex, gender (identity/presentation), and sexual orientation and race, ethnicity and racism.

Reporting on sex and gender	n/a
Reporting on race, ethnicity, or other socially relevant groupings	n/a
Population characteristics	n/a
Recruitment	n/a
Ethics oversight	n/a

Note that full information on the approval of the study protocol must also be provided in the manuscript.

Field-specific reporting

Please select the one below that is the best fit for your research. If you are not sure, read the appropriate sections before making your selection.

☒ Life sciences ☐ Behavioural & social sciences ☐ Ecological, evolutionary & environmental sciences

For a reference copy of the document with all sections, see nature.com/documents/nr-reporting-summary-flat.pdf

Life sciences study design

All studies must disclose on these points even when the disclosure is negative.

Sample size	No statistical method was used to predetermine sample size. The goal of this study was to investigate technological differences rat her than biological effects. Common model organisms (HeLa) were selected to investigate the performance of DIA search methods in typical proteomics experiments.
Data exclusions	No data were excluded from the analyses.
Replication	At least triplicate measurements were used for benchmarks involving the number of identified peptides or proteins. For quantitative benchmarks at least five replicates were used. This approach provides good balance between measurement time and statistically robust data.
Randomization	Samples measured with different methods were queued in alternating order (method 1, method 2, method 1, method 2, ...) to minimize potential technical bias caused by factors like column degradation or mass spectrometer performance drift over time.
Blinding	No blinding was performed as the study was of technical nature and no observer bias is expected.

Reporting for specific materials, systems and methods

We require information from authors about some types of materials, experimental systems and methods used in many studies. Here, indicate whether each material, system or method listed is relevant to your study. If you are not sure if a list item applies to your research, read the appropriate section before selecting a response.

Materials & experimental systems

n/a	Involved in the study
☒	☐ Antibodies
☐	☒ Eukaryotic cell lines
☒	☐ Palaeontology and archaeology
☒	☐ Animals and other organisms
☒	☐ Clinical data
☒	☐ Dual use research of concern
☒	☐ Plants

Methods

n/a	Involved in the study
☒	☐ ChIP-seq
☒	☐ Flow cytometry
☒	☐ MRI-based neuroimaging

Eukaryotic cell lines

Policy information about cell lines and Sex and Gender in Research

Cell line source(s)	The cell line Hela was purchased from DSMZ (No. ACC 57, https://www.dsmz.de/collection/catalogue/details/culture/ACC-57) and originates from an epithelial cervix carcinoma of a 31-year-old woman in 1951.
Authentication	The cell line has not been authenticated in the course of this study.
Mycoplasma contamination	All cell lines tested negative for mycoplasma contamination.
Commonly misidentified lines (See ICLAC register)	No, these cell lines do not belang to the commonly misidentified cell lines.

Plants

Seed stocks	No plants where used in the study.
Novel plant genotypes	n/a
Authentication	n/a
